# Conserved Antagonization of Type I Interferon Signaling by *Arterivirus* GP5 Proteins

**DOI:** 10.3390/v16081240

**Published:** 2024-08-01

**Authors:** Rissar Siringo Ringo, Amonrat Choonnasard, Tamaki Okabayashi, Akatsuki Saito

**Affiliations:** 1 Department of Veterinary Science, Faculty of Agriculture, University of Miyazaki, Miyazaki 889-2192, Japan; rissar_siringo_ringo@med.miyazaki-u.ac.jp (R.S.R.); amonrat_choonnasard@med.miyazaki-u.ac.jp (A.C.); okbys81@cc.miyazaki-u.ac.jp (T.O.); 2 Graduate School of Medicine and Veterinary Medicine, University of Miyazaki, Miyazaki 889-1692, Japan; 3 Center for Animal Disease Control, University of Miyazaki, Miyazaki 889-2192, Japan

**Keywords:** *Arterivirus*, GP5 proteins, interferon β signaling pathway, IFN-stimulated genes, *Arterivirus* persistent infection

## Abstract

*Arteriviruses* can establish persistent infections in animals such as equids, pigs, nonhuman primates, rodents, and possums. Some *Arteriviruses* can even cause overt and severe diseases such as Equine Arteritis in horses and Porcine Reproductive and Respiratory Syndrome in pigs, leading to huge economic losses. *Arteriviruses* have evolved viral proteins to antagonize the host cell’s innate immune responses by inhibiting type I interferon (IFN) signaling, assisting viral evasion and persistent infection. So far, the role of the *Arterivirus* glycoprotein 5 (GP5) protein in IFN signaling inhibition remains unclear. Here, we investigated the inhibitory activity of 47 *Arterivirus* GP5 proteins derived from various hosts. We demonstrated that all GP5 proteins showed conserved activity for antagonizing TIR-domain-containing adapter proteins inducing interferon-β (TRIF)-mediated IFN-β signaling through TRIF degradation. In addition, *Arterivirus* GP5 proteins showed a conserved inhibitory activity against IFN-β signaling, induced by either pig or human TRIF. Furthermore, certain *Arterivirus* GP5 proteins could inhibit the induction of IFN-stimulated genes. These findings highlight the role of *Arterivirus* GP5 proteins in supporting persistent infection.

## 1. Introduction

*Arteriviruses* form a unique cluster of viruses within the *Arteriviridae* family, one of four recognized families under the *Nidovirales* order [1]. So far, there are six subfamilies (*Crocarterivirinae*, *Equarterivirinae*, *Heroarterivirinae*, *Simarterivirinae*, *Variarterivirinae*, and *Zealarterivirinae*) within the *Arterivirus* cluster, including 23 distinct species [2]. *Arterivirus* harbors positive-sense, single-stranded linear RNA genomes spanning 13–15 kilobases (kb) in length, comprising 10–11 open reading frames (ORFs), except for simarteviruses which have 15 ORFs [2,3]. Eight ORFs encode the envelope protein (E), five glycoproteins (GP2, GP3, GP4, GP5, and GP5a), the membrane protein (M), and the nucleocapsid protein (N) [1]. GP5 and M proteins are the two major viral envelope proteins, driving *Arterivirus* assembly and budding [4]. GP5 proteins are the predominant envelope glycoproteins and represent a crucial epitope for neutralizing antibodies [5,6].

Several *Arteriviruses* can cause overt, severe diseases, manifesting a range of clinical manifestations from subclinical to severe systemic diseases. Lactate dehydrogenase-elevating virus (LDV) causes elevated lactate dehydrogenase levels in mice without pathological consequences for the host [7], while equine arteritis virus (EAV) causes respiratory and reproductive disease in equids, leading to severe respiratory distress and abortion in pregnant mares [8]. Similar to EAV, the porcine reproductive and respiratory syndrome virus (PRRSV) causes severe respiratory distress and abortion in pigs, and is mostly recognized due to its significant economic impact on the swine industry [9]. Simian hemorrhagic fever virus (SHFV) infection leads to a deadly hemorrhagic fever in macaques [10], whereas wobbly possum disease virus induces a fatal neurological syndrome in the Australian brushtail possum [11]. In addition, the *Arterivirus* can establish a persistent infection within the infected animals: LDV often persists in mice without inducing clinical signs, EAV can establish lifelong persistence in horses, PRRSV can persist in pigs for up to five months, and SHFV can persist for >10 years in baboons [12,13,14,15]. The persistence of an *Arterivirus* infection is attributed to immune evasion strategies, primarily through the inhibition of type I Interferon (IFN) signaling via multiple pathways [16].

The innate immune system is the initial defense against viral infections, activating pattern recognition receptors (PRRs); type I IFNs (IFN-α/β) being the most potent element of innate immunity against viruses [17]. Toll-like receptors (TLRs) were the first PRRs discovered in the innate immune system, playing a crucial role in triggering the inflammatory response [18]. Among all TLRs, TLR3 recognizes viral double-stranded RNA (dsRNA) and plays a pivotal role in antiviral immune responses by promoting the production of both type I IFN and inflammatory cytokines [19]. TLR3 stimulation by dsRNA leads to the recruitment of Toll–interleukin 1 receptor domain-containing adapter inducing interferon-β (TRIF), subsequently activating the TANK-binding kinase 1 (TBK1) to phosphorylate IFN regulatory factor 3 (IRF3), finally inducing the production of type I IFNs [20]. Subsequently, type I IFNs bind to their receptor, the IFN alpha and beta receptor subunit (IFNAR), activating the IFN-stimulated gene factor 3 (ISGF3) complex. Next, ISGF3 binds to IFN-stimulated response elements in gene promoters, inducing IFN-stimulated genes (ISGs) such as myxovirus resistance 1 (*Mx1*) and interferon-stimulated gene 15 (*Isg15*) [21]. These ISGs exhibit antiviral activity by inhibiting various stages of viral entry and replication [21,22,23]. Numerous *Arterivirus* proteins inhibit IFN downstream signaling. Previous studies demonstrated that nonstructural proteins 1 (nsp1), nsp2, nsp4, nsp7, and nsp11, M, and N proteins had inhibitory effects on IFN-β signaling and ISG induction [16,24,25,26,27]. However, the role of *Arterivirus* GP5 proteins in inhibiting IFN-β signaling and induction of ISGs remains to be elucidated.

In the present study, we investigated the function of 47 genetically diverse *Arterivirus* GP5 proteins in IFN-β signaling. Our study showed the conserved functions of *Arterivirus* GP5 proteins in inhibiting TRIF-mediated IFN-β signaling. Additionally, we discovered that various *Arterivirus* GP5 proteins have different effects on inhibiting ISG induction. These results suggest that *Arterivirus* GP5 proteins may play a crucial role in evading the host’s innate immune responses and promoting persistent viral infection.

## 2. Materials and Methods

### 2.1. Plasmids

cDNA sequences encoding 47 GP5 proteins with N-terminal HA tags were synthesized (Twist Bioscience, South San Francisco, CA, USA) by applying codon optimization. Details on the synthesized DNA sequences are provided in Supplementary Table S1. The cDNA inserts were cloned into a pCAGGS vector [28] pre-digested with AgeI-HF (New England Biolabs (NEB), Ipswich, MA, USA, Cat# R3552M) and NheI-HF (NEB, Cat# R3131M), using the In-Fusion Snap Assembly Master Mix (TaKaRa, Kusatsu, Japan, Cat# Z8947N). Plasmids were then amplified using NEB 5-alpha F′Iq Competent *Escherichia coli* (High Efficiency) (NEB, Cat# C2992H) and isolated with the PureYield Plasmid Miniprep System (Promega, Madison, WI, USA, Cat# A1222). The sequences of all plasmids were confirmed using the SupreDye v3.1 Cycle Sequencing Kit (M&S TechnoSystems, Osaka, Japan, Cat# 063001) with a Spectrum Compact CE System (Promega, Madison, WI, USA, Cat# A1222).

The IFN-Beta_pGL3 plasmid was gifted by Nicolas Manel (Addgene plasmid #102597; http://n2t.net/addgene:102597, accessed on 20 March 2024; RRID: Addgene_102597) [29]. The pCAGGS vector encoding human TRIF protein with a Myc tag was previously generated [30] and the human V5-IRF3-pcDNA3 was a gift from Saumen Sarkar (Addgene plasmid # 32713; http://n2t.net/addgene:32713, accessed on 20 March 2024; RRID:Addgene_32713) [31]. The pRL-TK plasmid is commercially available (Promega, Cat# E2241).

### 2.2. Construction of Plasmids Encoding Myc-Tagged Pig TRIF Protein

To generate a pCAGGS vector encoding the pig TRIF protein with a Myc tag, the cDNA sequences of pig TRIF proteins were synthesized (Twist Bioscience) by applying codon optimization. The synthesized DNA sequences are detailed in Supplementary Table S2. An insert containing the pig TRIF cDNA was cloned into a pCAGGS vector pre-digested with EcoRI-HF (NEB, Cat# R3101M) and NheI-HF using the NEBuilder HiFi DNA Assembly Master Mix (NEB, Cat# E2621F). Amplified PCR fragments encoding pig TRIF were cloned into the pCAGGS vector as described in Section 2.1. Plasmids were subsequently verified by sequencing.

### 2.3. Cell Culture

Lenti-X 293T cells (TaKaRa, Cat# Z2180N) and SK-6 cells [32] were maintained in Dulbecco’s modified Eagle medium (Nacalai Tesque, Kyoto, Japan, Cat# 08458-16), supplemented with 10% fetal bovine serum (Cytiva, Shinjuku-Ku, Japan, Cat# SH30396) and 1x penicillin–streptomycin (Pe/St, Nacalai Tesque, Cat# 09367-34) at 37 °C in a humidified incubator with 5% CO_2_.

### 2.4. Western Blotting

Lenti-X 293T cells were seeded into a 24-well plate (Fujifilm, Osaka, Japan, Cat# 630-28441) at 1.25 × 10^5^ cells per well, cultured overnight, and transfected with 12.5 ng of IFN-Beta_pGL3 plasmid, 225 ng of pRL-TK, 12.5 ng of pCAGGS-Myc-pigTRIF, and 250 ng of pCAGGS plasmids encoding HA-tagged GP5 proteins or pCAGGS empty plasmid. At 24 h after transfection, transfected cells were collected and lysed with 2× Bolt LDS sample buffer (Thermo Fisher Scientific, Cat# B0008) containing 2% β-mercaptoethanol (Bio-Rad, Hercules, CA, USA, Cat# 1610710) and incubated at 70 °C for 10 min. The expression of HA-tagged GP5 proteins was analyzed using Simple Western Abby (ProteinSimple, San Jose, CA, USA) with an anti-HA Tag (6E2) mouse monoclonal antibody (CST, Danvers, MA, USA, Cat# 2367S, ×200) and an Anti-Mouse Detection Module (ProteinSimple, Cat# DM-002). The total amount of input protein was quantified using a Total Protein Detection Module (ProteinSimple, Cat# DM-TP01).

### 2.5. IFN-β Luciferase Reporter Assay

Lenti-X 293T cells were seeded into a 96-well plate (Fujifilm, Cat# 635-28511) at 3 × 10^4^ cells per well. After culturing overnight, cells were transfected with the following combinations: (1) 2.5 ng of IFN-Beta_pGL3 plasmid, 45 ng of pRL-TK, 2.5 ng of pCAGGS-Myc-pigTRIF, and 50 ng of pCAGGS plasmids encoding HA-tagged GP5 proteins or pCAGGS empty plasmid, or (2) 5 ng of IFN-Beta_pGL3 plasmid, 40 ng of pRL-TK, 25 ng of Human V5-IRF3-pcDNA3, and 30 ng of pCAGGS plasmids encoding HA-tagged GP5 proteins or pCAGGS empty plasmid. Transfections were performed using the TransIT-LT1 Transfection Reagent (TaKaRa, Cat# V2304T) in Opti-MEM (Thermo Fisher Scientific, Minoto-Ku, Japan, Cat# 31985062). At 24 h after transfection, luciferase activity was measured using the Dual-Glo Luciferase Assay System (Promega, Cat# E2920). Firefly luciferase activity was normalized to that of Renilla luciferase. The relative activity (%) was calculated by comparing normalized luciferase values of cells co-transfected with *Arterivirus* GP5 protein plasmids to those of cells co-transfected with a pCAGGS empty plasmid. The assays were repeated ≥3 times. Data show the mean ± SD from one representative experiment.

### 2.6. TRIF Degradation Assay

Lenti-X 293T cells were seeded into 24-well plates (Fujifilm, Cat# 630-28441) at 1.25 × 10^5^ cells per well. Cells were cultured overnight and co-transfected with 250 ng of pCAGGS plasmid encoding HA-tagged GP5 proteins and 250 ng of pCAGGS plasmid encoding the Myc-tagged pig TRIF protein. Cellular lysates were prepared as described above. The expression of Myc-tagged TRIF protein was quantified using an anti-Myc Tag (9B11) mouse monoclonal antibody (CST, Cat# 2276S, ×100) and an Anti-Mouse Detection Module. The amount of input protein was determined using a Total Protein Detection Module, as described above.

### 2.7. Measurement of IFN-Stimulated Gene Expression

To measure ISG induction by poly (I:C), SK-6 cells were seeded in sextuplicate at 1 × 10^4^ cells per well in a 96-well plate. Then, cells were transfected with 50 ng/mL poly (I:C) (Sigma-Aldrich, St. Louis, MO, USA, Cat# P1530) and 50 ng of pCAGGS plasmids encoding HA-tagged GP5 proteins or a pCAGGS empty plasmid. Transfections were performed using the TransIT-X2 Dynamic Delivery System (TaKaRa, Cat# V6100) according to manufacturer’s instructions. After 24 h of incubation, total RNA was extracted using the CellAmp Direct RNA Prep Kit for RT-PCR (Real Time) (TaKaRa, Cat# 3732) according to manufacturer’s instructions.

To measure the ISG induction triggered by IFN-β, SK-6 cells were seeded and transfected with 50 ng of pCAGGS plasmids encoding HA-tagged GP5 proteins or a pCAGGS empty plasmid, as described previously. After overnight incubation, the cells were treated with 100 ng/mL pig IFN-β (Kingfisher Biotech, St. Paul, MN, USA, Cat# RP0011S-025). After 24 h of incubation, the *Mx1* and *Isg15* mRNA levels were quantified by a qRT-PCR assay using the One Step TB Green PrimeScript PLUS RT-PCR Kit (Perfect Real Time) (TaKaRa, Cat# RR096A). The PCR cycling conditions were as follows: 42 °C for 5 min, 95 °C for 10 s, followed by 40 cycles of 95 °C for 5 s and 60 °C for 34 s. The expression levels of *Mx1* and *Isg15* mRNA were normalized to those of porcine β-actin, which served as an endogenous control, using the ΔΔCt method. The specific sequence primers for ISG mRNA levels were previously described [33], and information on primer sets is provided in Supplementary Table S3.

### 2.8. Alignment of GP5 Amino Acids and Phylogenetic Analysis

We aligned GP5 proteins from 47 *Arterivirus* strains utilizing the MUSCLE algorithm within the MEGA X (version 11.0.13) software. Subsequently, we generated a phylogenetic tree based on the aligned amino acid sequences sourced from public databases. Evolutionary analysis was carried out using maximum likelihood and neighbor-joining methods, utilizing the Jones–Taylor–Thornton matrix-based model, with 1000 bootstrap replicates for robustness assessment.

### 2.9. Statistical Analysis

The results are expressed as the mean and standard deviation of six measurements from one assay, representing ≥2–3 independent experiments. Differences in relative values between the *Arterivirus* GP5 proteins and the empty plasmid were examined using a one-way ANOVA followed by Dunnett’s multiple comparisons test. A *p* ≤ 0.05 was considered statistically significant. Differences in the relative value of the IFN-β luciferase reporter assay induced by pig TRIF versus human TRIF were analyzed using a two-way ANOVA, followed by Šídák’s multiple comparisons test. All statistical analyses were performed using Prism 10 software v10.2.1 for Windows (GraphPad Software, Boston, MA, USA).

## 3. Results

### 3.1. Genetic Characteristic of Arterivirus GP5 Proteins

**Figure 1:**
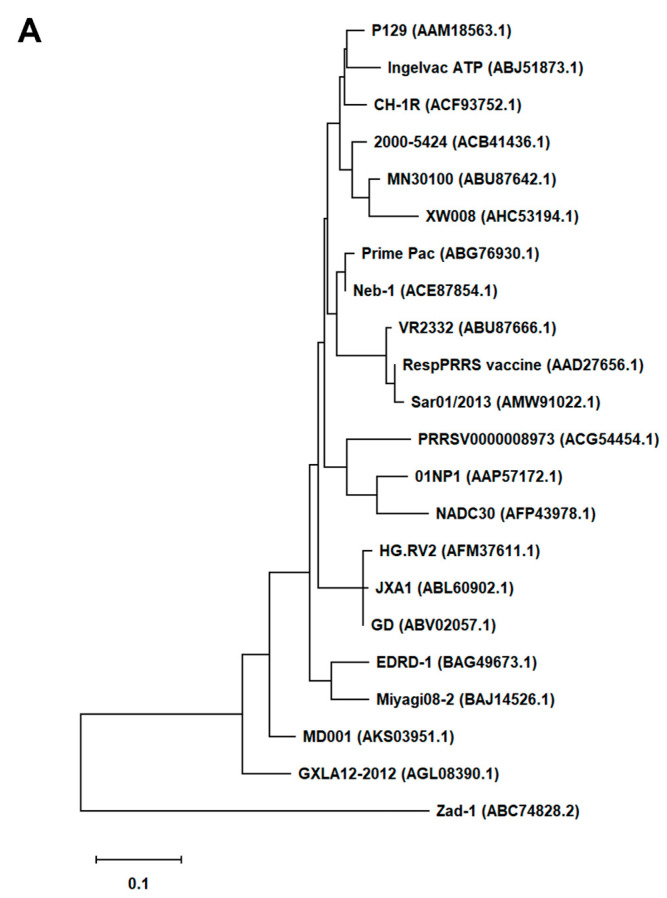

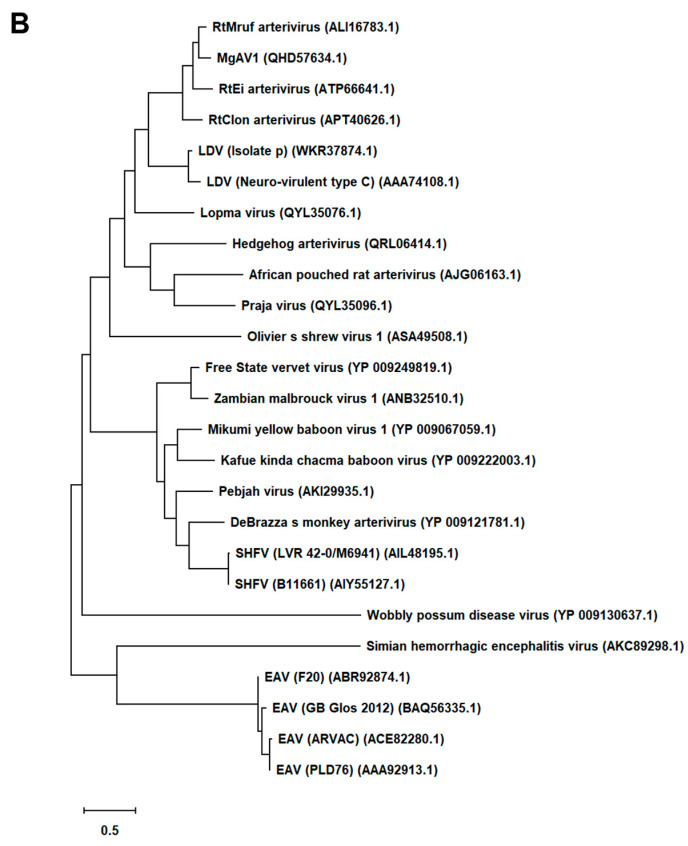
Phylogenetic tree of *Arterivirus* GP5 proteins. (**A**) The phylogenetic tree of PRRSV GP5 proteins was generated using sequences obtained from public databases. (**B**) The phylogenetic tree of other *Arterivirus* GP5 proteins was constructed using the MEGA software, and the evolutionary analysis was conducted using the maximum likelihood and neighbor-joining methods based on the Jones–Taylor–Thornton matrix-based model.

First, we generated an alignment of the amino acid sequence of 47 *Arterivirus* GP5 proteins, including 22 strains of PRRSV (Figure 1A) and 25 strains from other *Arteriviruses* (Figure 1B). While the Zad-1 strain belongs to PRRSV-1, the other strains belong to PRRSV-2. The GP5 proteins from 20 PRRSV strains contain 200 amino acids (aa), whereas 01NP1 and Zad-1 contain 199 and 201 aa, respectively. The other *Arteriviruses* comprise four strains of EAV, three of LDV, two of SHFV, and one from the other *Arterivirus* species. The length of GP5 proteins varies significantly among strains of the other *Arteriviruses*. For instance, the EAV GP5 protein comprises 255 aa, while SHFV GP5 encompasses 278 aa.

In our phylogenetic analysis, we calculated the sequence identity and similarity of *Arterivirus* GP5 proteins (Figure S1A–D). Among 21 strains of PRRSV-2, the percentage of similarity in their identities (PID) ranged from 80.5% to 99.5%. Notably, when comparing these strains with PRRSV-1 (specifically Zad-1), the observed PID between the two groups was 53.6%. Furthermore, the similarity between PRRSV-2 and PRRSV-1 (Zad-1) was 65.8%. For other *Arterivirus* GP5 proteins, the PID and similarity ranged from 8.3% to 99.6% and 15.6% to 99.6%, respectively. When ≥2 aa sequences show 100% PID and 100% similarity, the aa in the sequences compared match exactly and have similar physicochemical properties, such as charge, size, and hydrophobicity. Furthermore, it suggests a very close relationship between the sequences. However, the PID and similarity among *Arterivirus* species were only 27.1% and 40.6%, respectively.

### 3.2. GP5 Proteins from Both PRRSV-2 (GD) and PRRSV-1 (Zad-1) Inhibit TRIF-Mediated IFN-β Signaling

**Figure 2:**
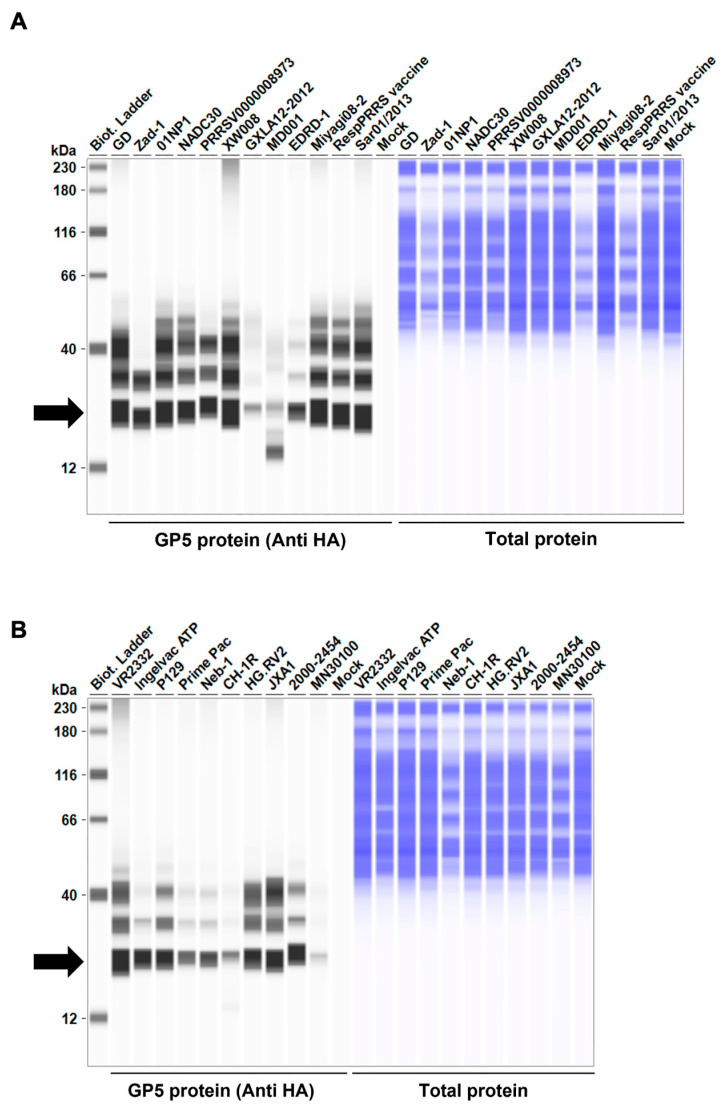

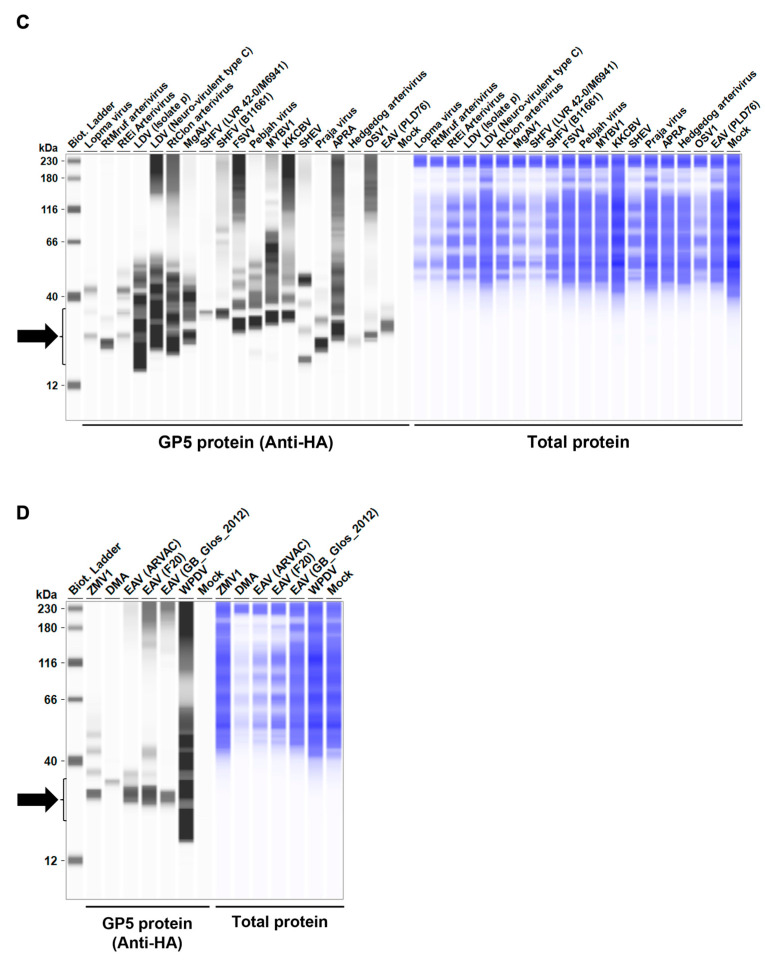
*Arterivirus* GP5 protein expression levels in Lenti-X 293T cells. (**A**–**D**) The expected HA-tagged GP5 protein size ranged between 18.49 and 32.54 kDa, according to the Protein Molecular Weight website (https://www.bioinformatics.org/sms/prot_mw.html, accessed on 28 March 2024). The black arrows indicate the size of HA-tagged GP5 proteins.

Before testing the inhibitory effect of *Arterivirus* GP5 proteins on IFN-β signaling, we evaluated the expression levels of HA-tagged *Arterivirus* GP5 proteins in Lenti-X 293T cells. We observed a comparable expression of GP5 proteins in transfected cells (Figure 2A–D). Subsequently, before assessing the inhibitory potential of *Arterivirus* GP5 proteins, we verified that co-transfection of IFN-β Luc plasmid with pig or human TRIF plasmids significantly induced Firefly luciferase (Figure S2A).

**Figure 3:**
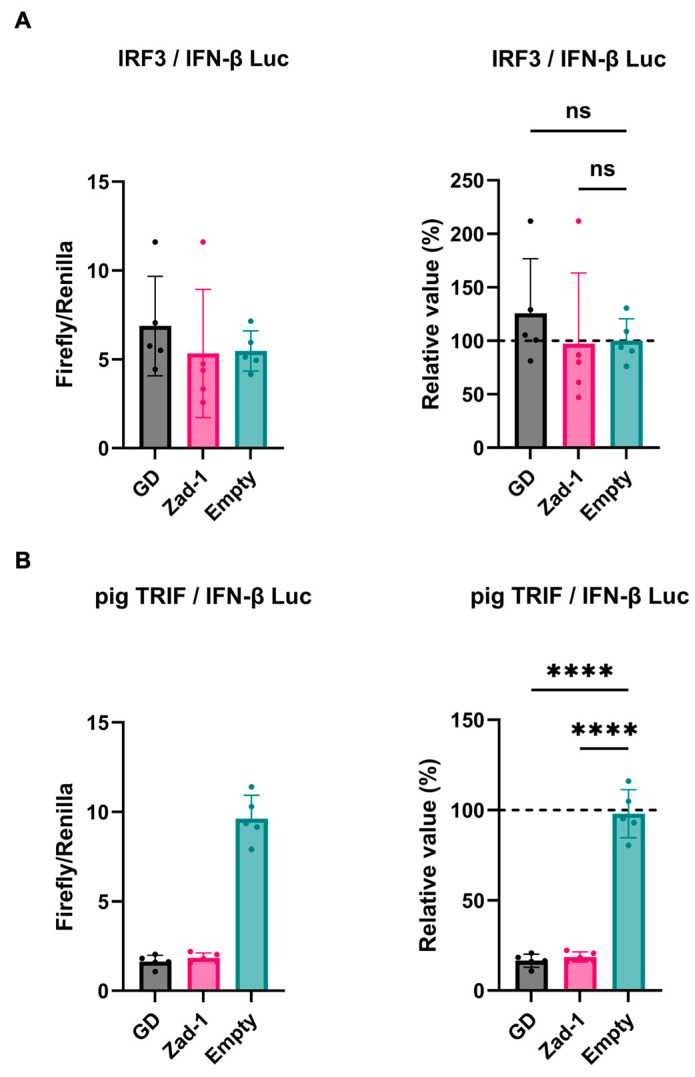
Different effects of PRRSV GP5 proteins on IFN-β signaling induced by IRF3 or pig TRIF. (**A**) Inhibitory effect of PRRSV-2 (GD) and PRRSV-1 (Zad-1) GP5 proteins on cells co-transfected with IFN-Beta_pGL3 and Human V5-IRF3-pcDNA3 plasmids. (**B**) Inhibitory effect of PRRSV-2 (GD) and PRRSV-1 (Zad-1) GP5 proteins on cells co-transfected with IFN-Beta_pGL3 and pCAGGS-Myc-pigTRIF plasmids. Differences between cells transfected with PRRSV-2, PRRSV-1 protein GP5 plasmids, or an empty plasmid were examined by one-way ANOVA followed by Dunnett’s multiple comparison test. **** *p* < 0.0001 and ns (not significant).

Next, we investigated the inhibitory potential of PRRSV-2 (GD) and PRRSV-1 (Zad-1) GP5 proteins on IFN-β signaling by co-transfecting Lenti-X 293T cells with GP5 protein plasmids. We found that GP5 proteins did not show a significant effect on IRF3-mediated IFN-β signaling (Figure 3A); however, GP5 proteins significantly inhibited TRIF-mediated IFN-β signaling (Figure 3B).

### 3.3. GP5 Proteins of Diverse Arteriviruses Inhibit Pig TRIF-Mediated IFN-β Signaling

**Figure 4:**
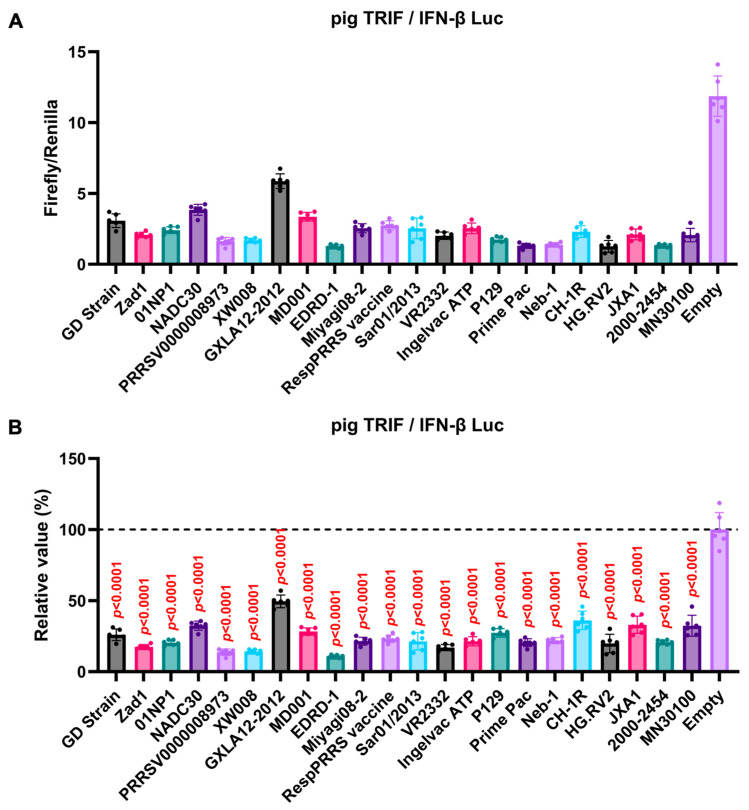

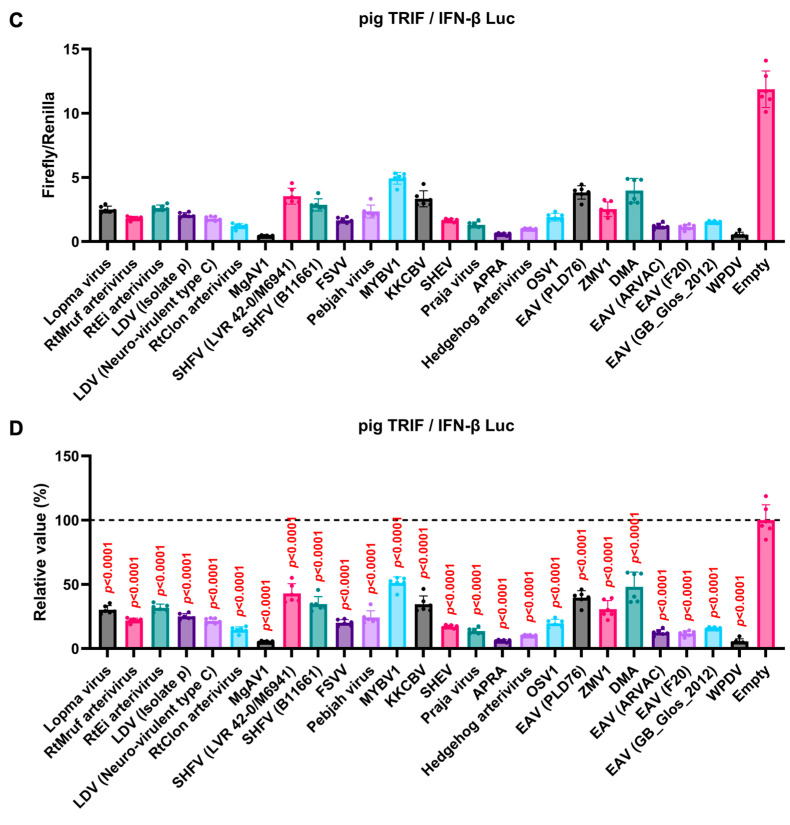
Inhibitory effect of *Arterivirus* GP5 proteins on pig TRIF-mediated IFN-β signaling. (**A**,**C**) Raw data of the luciferase reporter assay. The RLU of Firefly luciferase was divided by the RLU of Renilla luciferase. (**B**,**D**) Relative value of IFN-β luciferase reporter assay. Differences between cells transfected with plasmids expressing *Arterivirus* GP5 proteins or an empty plasmid were examined by one-way ANOVA followed by Dunnett’s multiple comparison test.

We observed an inhibitory effect of PRRSV-2 (GD) and PRRSV-1 (Zad-1) GP5 proteins on IFN-β signaling (Figure 3B). To investigate whether other *Arterivirus* GP5 proteins have a similar inhibitory effect on TRIF-mediated IFN-β signaling, 47 *Arterivirus* GP5 protein plasmids were used to co-transfect Lenti-X 293T cells with IFN-β Luc and pig TRIF plasmids (Figure 4A,B). All GP5 proteins inhibited pig TRIF-mediated IFN-β signaling, thus demonstrating that IFN-β signaling inhibition is conserved across *Arterivirus* GP5 proteins. Further, we examined the dose-dependent inhibitory effects of *Arterivirus* GP5 proteins on TRIF-mediated IFN-β signaling. Our results revealed that increasing dose of GP5 protein plasmids enhanced their inhibitory impact on TRIF-mediated IFN-β signaling (Figure S3A,B), reinforcing our hypothesis that GP5 proteins can inhibit IFN-β signaling in a dose-dependent manner.

### 3.4. GP5 Proteins of Diverse Arteriviruses Inhibit the Human TRIF-Mediated IFN-β Signaling Pathway

**Figure 5:**
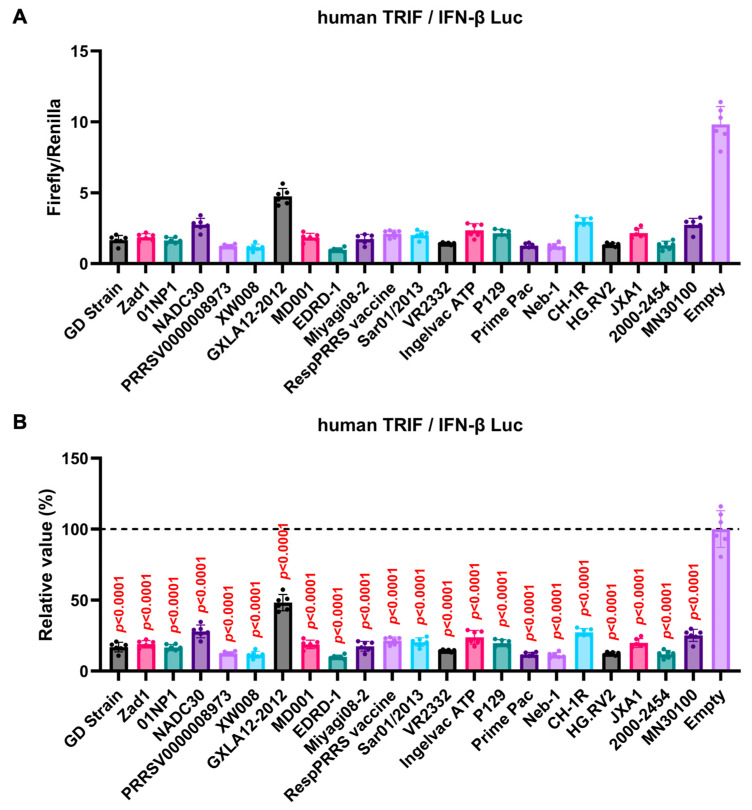

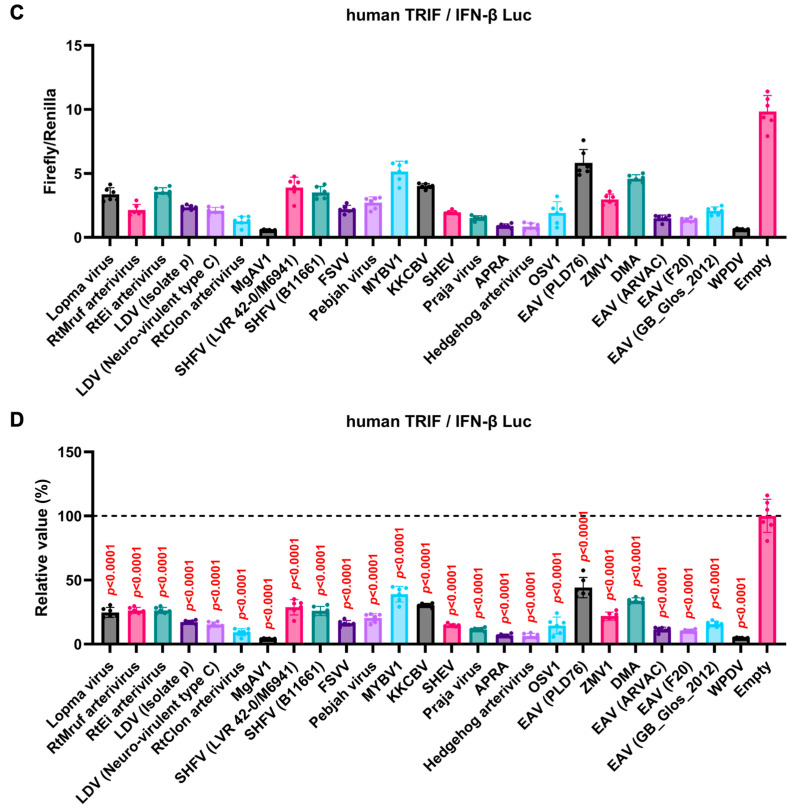
Inhibitory effect of *Arterivirus* GP5 proteins on human TRIF-mediated IFN-β signaling. (**A**,**C**) Raw data of the luciferase reporter assay. The RLU of Firefly luciferase was divided by that of Renilla luciferase. (**B**,**D**) Relative value in the IFN-β luciferase reporter assay. Differences between cells transfected with plasmids expressing *Arterivirus* GP5 proteins or an empty plasmid as examined by one-way ANOVA followed by Dunnett’s multiple comparison test.

**Figure 6:**
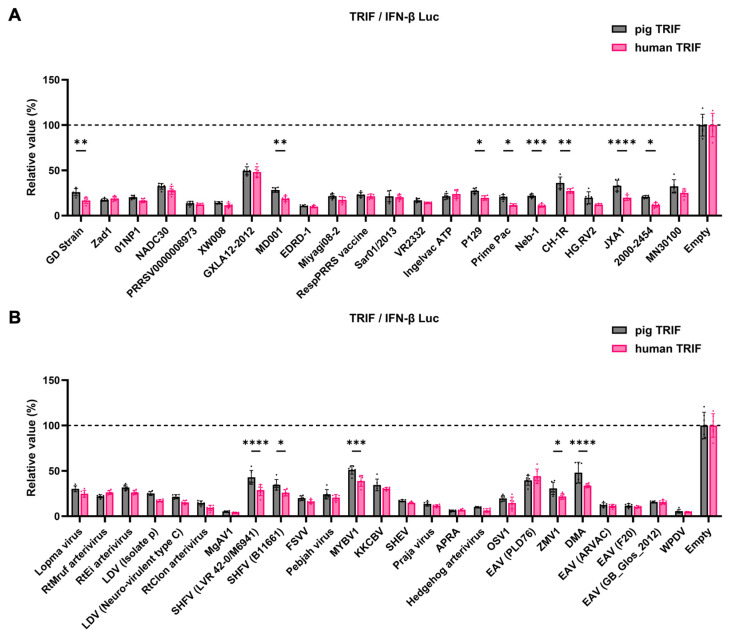
Inhibitory effect of *Arterivirus* GP5 proteins on IFN-β signaling induced by pig or human TRIF. (**A**) Inhibitory effect of PRRSV GP5 proteins on IFN-β signaling induced by pig or human TRIF. (**B**) Inhibitory effect of other GP5 proteins on IFN-β signaling induced by pig or human TRIF. Differences in inhibitory effects on pig and human TRIF as examined by two-way ANOVA followed by Šídák’s multiple comparisons test. **** *p* < 0.0001, *** *p* < 0.001, ** *p* < 0.01, and * *p* < 0.05.

The GP5 proteins showed a conserved inhibitory activity over the pig TRIF-mediated IFN-β signaling (Figure 4A,B). To examine whether *Arterivirus* GP5 proteins have the same effect as human TRIF, we tested 47 *Arterivirus* GP5 protein plasmids for co-transfection with the IFN-β Luc and human TRIF plasmids in Lenti-X 293T cells (Figure 5A,B). *Arterivirus* GP5 proteins inhibited human TRIF-mediated IFN-β signaling, suggesting that *Arterivirus* GP5 proteins possess a conserved capability to counteract the IFN-β signaling induced by both pig and human TRIF (Figure 6).

### 3.5. GP5 Proteins of Various Arteriviruses Have a Conserved Degradation Activity for TRIF

**Figure 7:**
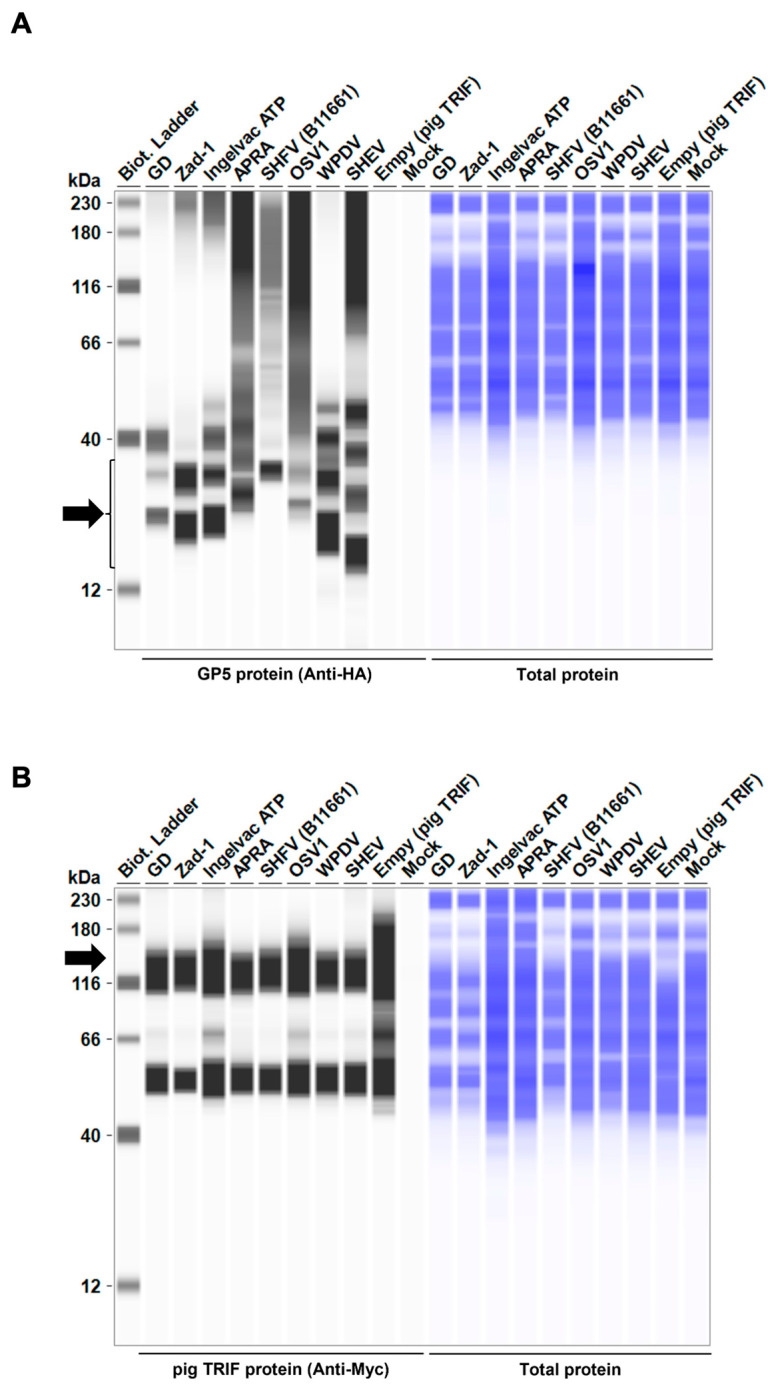

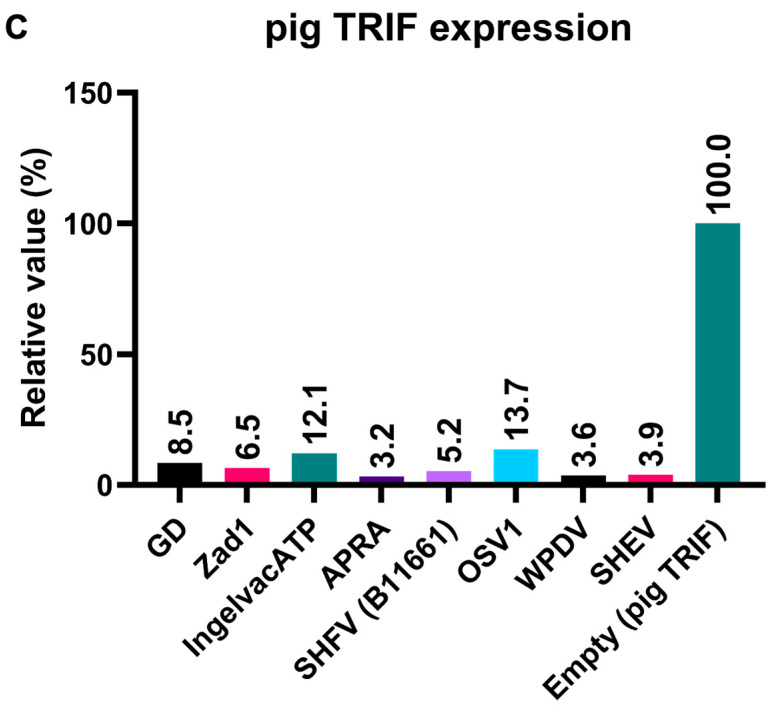
*Arterivirus* GP5 proteins degrade pig TRIF protein. (**A**) Expression levels of HA-tagged *Arterivirus* GP5 proteins in transfected Lenti-X 293T cells. The expected HA-tagged GP5 protein size was 18.49–32.53 kDa, as indicated by a black arrow. (**B**) Myc-tagged pig TRIF expression levels in transfected Lenti-X 293T cells. The expected Myc-tagged pig TRIF size was 129 kDa, as indicated by a black arrow. (**C**) Relative pig TRIF expression levels in the presence of *Arterivirus* GP5 proteins. Relative value calculated from the corrected area of the TRIF band with Compass for Simple Western software version 6.3.0.

To elucidate the mechanism underlying the inhibitory effect of *Arterivirus* GP5 proteins on TRIF-mediated IFN-β signaling, we performed Western blotting to examine pig TRIF levels in the presence of *Arterivirus* GP5 proteins (Figure 7A,B). The expression of GP5 proteins resulted in an 86–96% degradation of pig TRIF (Figure 7C). Our results suggest that *Arterivirus* GP5 proteins have a conserved function in degrading pig TRIF protein to inhibit TRIF-mediated IFN-β signaling.

### 3.6. Arterivirus GP5 Proteins Differently Inhibit Poly (I:C) and IFN-β Triggered ISG Induction

**Figure 8:**
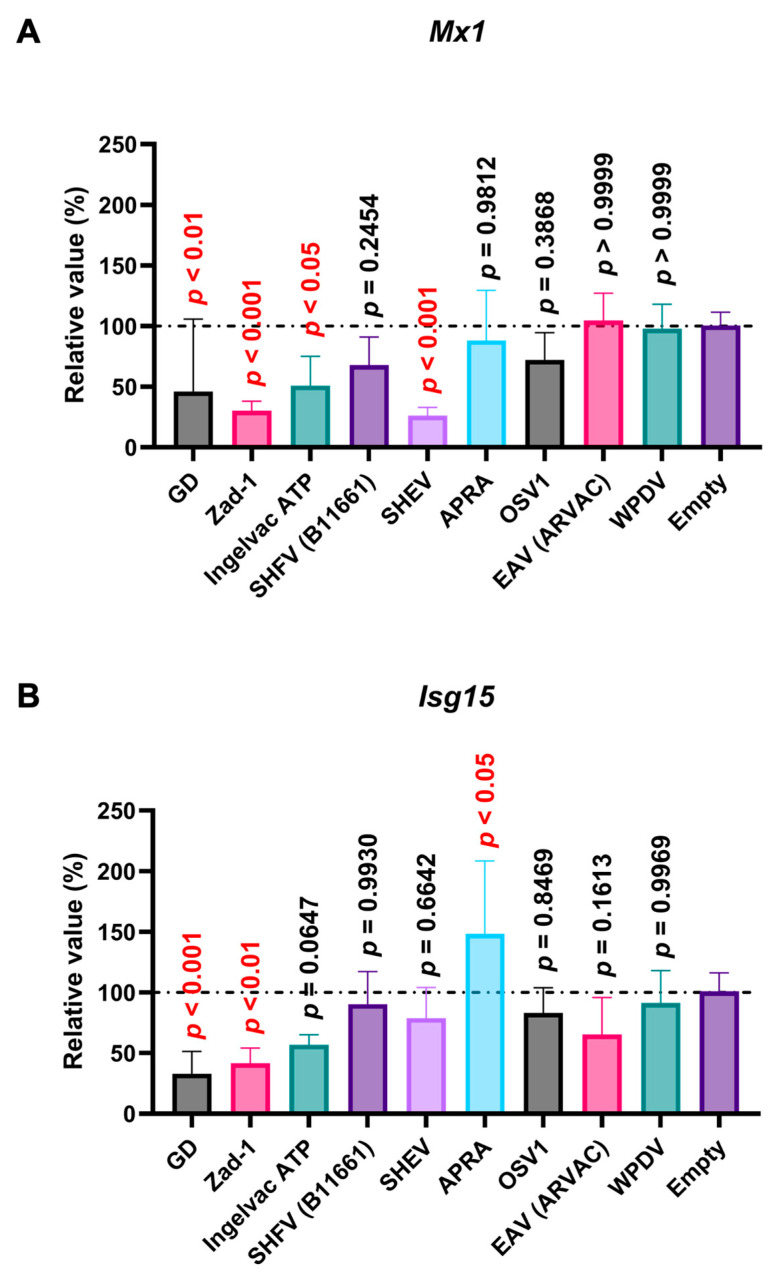
*Arterivirus* GP5 proteins inhibit ISG mRNA induction in SK-6 cells treated with poly (I:C). The results show the mean and standard deviation of sextuplicate measurements from one assay. (**A**) Impact of *Arterivirus* GP5 proteins on *Mx1* induction. (**B**) Impact of *Arterivirus* GP5 proteins on *Isg15* induction. Differences between cells transfected with an empty plasmid and plasmids expressing *Arterivirus* GP5 proteins as examined by one-way ANOVA followed by Dunnett’s multiple comparison test.

Poly (I:C), a potent IFN inducer that mimics double-stranded viral RNA, is recognized by TLR3, Retinoic Acid-Inducible Gene I (RIG-I), and Melanoma Differentiation-Associated protein 5 (MDA5) [34]. The interaction of poly (I:C) with TLR3 and RIG-I/MDA5 stimulates the production of type I IFNs [34,35], leading to the induction of ISGs such as *Mx1* and *Isg15*. Before assessing the effect of *Arterivirus* GP5 proteins on poly (I:C)-triggered ISGs induction, we verified that treating SK-6 cells with poly (I:C) efficiently induced ISG (Figure S4A). We found that PRRSV-2 (GD) and PRRSV-1 (Zad-1) GP5 proteins significantly inhibited *Mx1* and *Isg15* induction upon poly (I:C) treatment (Figure 8A,B). Additionally, we observed a similar inhibitory effect with the GP5 proteins of PRRSV-2 (Ingelvac ATP) and simian hemorrhagic encephalitis virus (SHEV) on *Mx 1* induction (Figure 8A).

**Figure 9:**
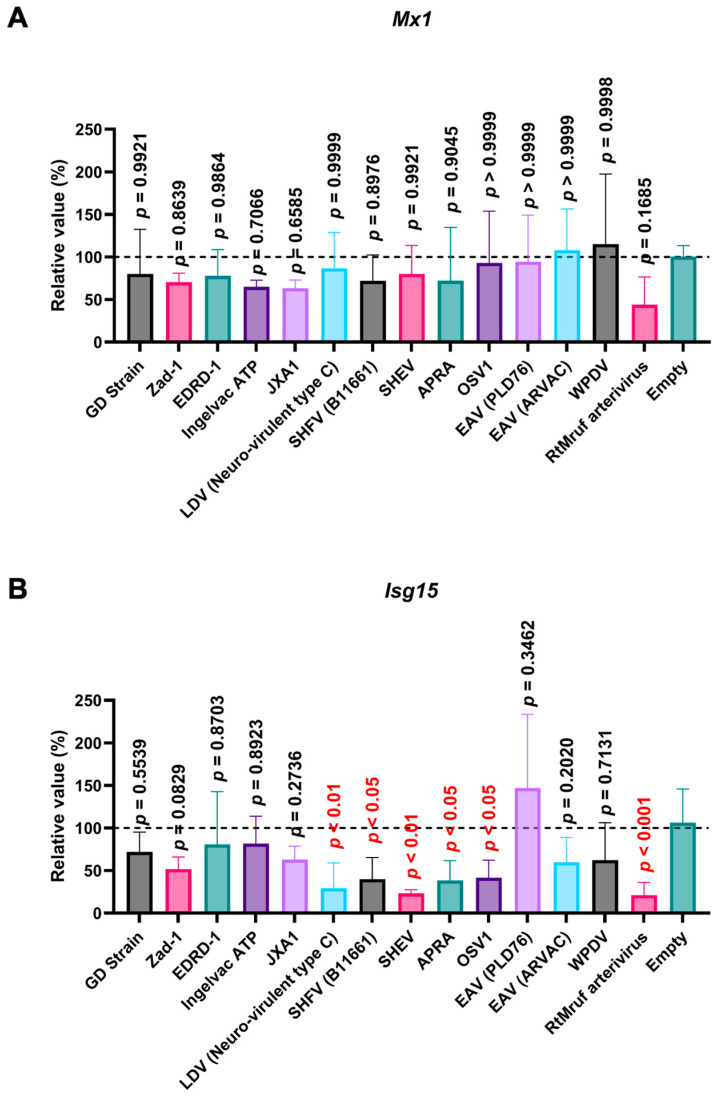
*Arterivirus* GP5 proteins inhibit ISG mRNA induction in SK-6 cells treated with IFN-β. The results show the mean and standard deviation of sextuplicate measurements from one assay. (**A**) Impact of *Arterivirus* GP5 proteins on *Mx1* induction. (**B**) Impact of *Arterivirus* GP5 proteins on *Isg15* induction. Differences between cells transfected with an empty plasmid and plasmids expressing *Arterivirus* GP5 proteins as examined by one-way ANOVA followed by Dunnett’s multiple comparison test.

Next, we examined the impact of *Arterivirus* GP5 proteins on ISG induction in SK-6 cells treated with IFN-β. Before assessing the effect of *Arterivirus* GP5 proteins on IFN-β-triggered ISG induction, we confirmed a robust ISG induction in SK-6 cells upon IFN-β treatment (Figure S4B). GP5 proteins of RtMruf arterivirus, LDV (Neuro-virulent type C), SHFV (B11661), SHEV, African pouched rat arterivirus, and Oliver’s shrew virus 1 inhibited *Isg15* induction upon IFN-β treatment (Figure 9B), while showing no effect on *Mx1* (Figure 9A). These results highlight the different inhibitory capacities of *Arterivirus* GP5 proteins over ISG induction by poly (I:C) and IFN-β treatment.

## 4. Discussion

In this study, we showed that *Arterivirus* GP5 proteins inhibit TRIF-mediated IFN-β signaling through TRIF degradation. This inhibitory activity is conserved across 47 different *Arterivirus* GP5 proteins. However, GP5 proteins exhibit varying inhibitory capacities for ISG induction.

Reportedly, GP5 proteins can inhibit IRF3 phosphorylation [36]. In contrast, our study revealed that GP5 proteins from PRRSV-2 and PRRSV-1 (GD and Zad-1 strains) did not significantly affect IRF3-mediated IFN-β signaling (Figure 3A). This discrepancy may be attributed to methodological differences. Zhixuan et al. used poly (I:C) to induce IRF3 phosphorylation, suggesting that GP5 proteins may inhibit IRF3 phosphorylation by interacting with upstream signaling components or adapter complexes, such as TRIF or mitochondrial antiviral-signaling protein (MAVS), rather than by directly targeting the IRF3. Notably, GP5 proteins significantly inhibited pig TRIF-mediated IFN-β signaling (Figure 3B), supporting that GP5 proteins might prevent IRF3 phosphorylation by interacting with TRIF.

Moreover, the 47 *Arterivirus* GP5 proteins studied herein share a conserved inhibitory function on IFN-β signaling induced by pig TRIF (Figure 4B,D). Additionally, our results demonstrate that eight phylogenetically diverse *Arterivirus* GP5 proteins can degrade pig TRIF (Figure 7A), suggesting that *Arterivirus* GP5 proteins have a conserved function, degrading pig TRIF to inhibit TRIF-mediated IFN-β signaling. Although several *Arterivirus* envelope proteins—nsp1, nsp2, nsp4, nsp11, M protein, and N protein—and suppress IFN-β responses for evasion from the innate immune system [16,24,25,26,37,38,39,40,41], none appear to interact with TRIF. Our preliminary findings offer insights into the interaction between *Arterivirus* GP5 proteins and TRIF, deepening our understanding of their potential role in facilitating virus evasion from host immune responses and maintaining persistent infection.

Notably, GP5 proteins effectively inhibited human TRIF-mediated IFN-β signaling, indicating a conserved function for 47 *Arterivirus* GP5 proteins (Figure 5B,D). Interestingly, certain GP5 protein had a significantly stronger inhibitory effect on IFN-β signaling with human TRIF than pig TRIF (Figure 6A,B), even when the human TRIF induced IFN-β production at tenfold higher levels than the pig TRIF (Figure S2A,B). Additionally, human and pig TRIF share only 71.6% PID (https://www.uniprot.org/uniprotkb, accessed on 19 June 2024). These findings suggest that *Arterivirus* may evolve into zoonotic pathogens. Accordingly, a previous study demonstrated that SHFV can replicate in human monocytes [42].

In contrast to the conserved inhibitory function of the 47 *Arterivirus* GP5 proteins, *Arterivirus* GP5 proteins differently inhibited ISG induction upon poly (I:C) treatment. Our results demonstrated that the GP5 proteins of PRRSV-2 (GD and Ingelvac ATP), PRRSV-1 (Zad-1), and SHEV could inhibit *Mx1* induction, while Ingelvac ATP and SHEV failed to inhibit *Isg15* induction (Figure 8A,B). This variability could be attributed to variations in the aa sequences among *Arterivirus* GP5 proteins, resulting in differential interactions with the regulatory elements of *Mx1* or *Isg15*. Moreover, PRRSV GP5 proteins failed to inhibit ISG induction in cells treated with IFN-β (Figure 9A,B). This discrepancy might be attributed to the different mechanisms whereby poly (I:C) and IFN-β induce ISGs, leading to variations in the degree and spectrum of their inhibitory effects. Notably, one *Arterivirus* GP5 protein (SHEV strain) inhibited ISG induction in SK-6 cells treated with either poly (I:C) or IFN-β. Furthermore, six *Arterivirus* GP5 proteins significantly inhibit *Isg15* induction but failed to inhibit *Mx1* induction (Figure 9A,B), also due to differential interactions with regulatory elements of *Mx1* or *Isg15*. To our knowledge, this is the first report on the role of *Arterivirus* GP5 proteins in inhibiting ISG induction.

*Arterivirus* (EAV, LDV, SHFV, and PRRSV) are well-known immunosuppressive or immunomodulatory agents in the host [43]. Our results depict that GP5 proteins aid *Arterivirus* to manipulate host immune responses by inhibiting TRIF-mediated IFN-β signaling and ISG induction. This ability to manipulate host immune responses may increase the host’s susceptibility to secondary infection by other pathogens. In fact, PRRSV—the most well-known *Arterivirus*—often leads to coinfection with other respiratory pathogens, such as swine influenza virus, porcine circovirus type 2, and *Mycoplasma hyopneumoniae*. Coinfections involving PRRSV significantly increase cytokine responses, leading to various clinical signs, pathological lesions, and death [44,45,46]. These coinfections pose substantial health challenges and contribute substantially to worldwide economic losses in pork production [44].

As a major structural protein, GP5 is the primary target of neutralizing antibodies induced by PRRSV vaccination or prior infection [47]. Moreover, GP5 proteins are crucial for eliciting humoral and cellular immune responses against PRRSV infection and proposed as excellent candidates for developing new vaccines [47,48,49]. Given its role in immune modulation and its potential for vaccine development, a better understanding GP5 proteins can lead to more effective strategies for controlling PRRSV and other *Arterivirus* infections as well as enhancing animal health and reducing economic loss.

This study has several limitations. First, a luciferase reporter system was used to explore the impact of *Arterivirus* GP5 proteins on TRIF-mediated IFN-β signaling in human-derived Lenti-X 293T cells instead of cells from natural hosts. In addition, the transfection of SK-6 cells with GP5 protein plasmids was utilized to assess the impact of *Arterivirus* GP5 proteins on ISG induction. Although these methods enable quantitative measurements, the experimental conditions may not completely reflect natural viral infection scenarios. in vivo, expression levels of viral proteins and the overall cellular environment can substantially differ from those observed in transfection systems.

Furthermore, the study focused on the overexpression of GP5 proteins. Since viral proteins often function contextually, studying a single viral protein may overlook important interactions or dependencies. In the future, we should explore whether *Arterivirus* GP5 proteins exert similar effects on molecules from other host species and validate our findings using recombinant viruses expressing various *Arterivirus* GP5 proteins.

In conclusion, our results revealed that *Arterivirus* GP5 proteins could inhibit TRIF-mediated IFN-β signaling through TRIF degradation, suggesting that this is a conserved function of *Arterivirus* GP5 proteins. Additionally, *Arterivirus* GP5 proteins differently inhibit ISG induction. These results deepen our understanding of the role of *Arterivirus* GP5 proteins in maintaining persistent and secondary infection, thereby contributing to the development of protective strategies. Investigations into the sequence of GP5 proteins to identify the specific domains and/or residues responsible for inhibiting the IFN-β signaling or ISG induction would be of further interest to deepen our understanding of the pathophysiological role of *Arterivirus* GP5 proteins.

## Data Availability

The original contributions presented in the study are included in the article/Supplementary Material, further inquiries can be directed to the corresponding author.

## Supplementary Materials

The following supporting information can be downloaded at: https://www.mdpi.com/article/10.3390/v16081240/s1, Figure S1: Sequence identity and similarity of *Arterivirus* GP5 proteins; Figure S2: Induction of Firefly luciferase; Figure S3: Dose-dependent inhibition of IFN-β signaling by *Arterivirus* GP5 proteins; Figure S4: IFN-stimulated genes (ISGs) induction triggered by Poly (I:C) or IFN-β; Table S1: Synthesized DNAs for generating plasmids encoding GP5 proteins; Table S2: Synthesized DNA for generating plasmids encoding pig TRIF protein; Table S3: The oligonucleotides used to quantify ISGs mRNA levels.
