# Supplementary material for: Conserved Antagonization of Type I Interferon Signaling by Arterivirus GP5 Proteins
Source: Viruses. 2024 Aug 1;16(8):1240. doi: 10.3390/v16081240 (PMC11358952; doi:10.3390/v16081240)
Supplement: Supplementary file 1 [file viruses-16-01240-s001.zip › 240701_Supplementary Tables.pdf]

viruses

MDPI

Supplementary Table S1. Synthesized DNAs for generating plasmids encoding GP5 proteins

PRRSV-2 (GD)<br>Accession# ABV02057.1	TGACTACGCGACCGGTTTGGGAAAATGCCTCACTGCGTGTTGCTGCTCAAGA<br>TTGCTGTTTCTTTGGTGTATCGTGCCCTTCTACCTGGCAGTCCTTGTGAACGCA<br>TCTAATAATAACAGCAGCCATATACAACTCATATACAATTTGACATTGTGTG<br>AGCTGAACGGGACTGACTGGCTCGCACAGAAATTTGATTGGGCTGTCGAAA<br>CGTTCGTGATTTTCCCAGTTCTTACGCATATTGTTAGCTATGGTGCCTTGACTA<br>CCTCACACTTTCTCGACACGGTCGGACTGGCCACCGTCTCTACGGCCGGATA<br>CTATCACGGCAGGTACGTCTTGTCTTCAATTTACGCGGTGTGTGCCCTCGCTG<br>CACTTATCTGTTTTGTTATTAGATTGGCAAAAAACTGTATGAGCTGGAGGTAC<br>TCTTGCACAAGGTACACCAATTTCCTCCTGGATACCAAGGGCCGCCTGTACC<br>GCTGGCGGTCACCTGTCATAGTCGAAAAAGGCGGCAAAGTGGAGGTCGAGG<br>GACATCTGATAGACCTTAAACGCGTCGTCCTGGACGGCAGTGCCGCAACTCC<br>GCTGACAAGGGTGTCAGCAGAGCAATGGGGACGGTTGTGAGCTAGCAGATC<br>TTTTT
PRRSV-1 (Zad-1)<br>Accession# ABC74828.2	TTTTGGCAAAGAATTCGCCACCATGAGAGATCATATGGTTTTGCATGAGTAC<br>GTGAACGCAGCTGGTATCACATGCACCGGTAGATGTAGTCGCACTTTGGGTC<br>AGCCTAGTACGCACCATTTTTATCTTTGGTGGCTTTTTTTGCTTTGCATAGGCC<br>TCTCCTGGTCATTTGCTGATGGGAATGGAAATAGCAGTACCTACCAATACAT<br>CTATGATCTTACGATATGTGAACTTAATGGCACCAACTGGTTGGCGTCACGC<br>TTCAGCTGGGTTGTGGAAAGTTTTGTGTTTTATCCTGTCGTCACTCACATTCTT<br>TCTCTCGGGTTCCTGACCACTAGTCATTTCTTCGATATGCTCGGTCTGGGTGC<br>GGTGGCTGCTGCAGGCTTTCATGGTCAGAGGTACGTCTTGTCATCTATATACG<br>GGATTAGCGCCCTGGCTGCATTCGTCTGTTTCGCAATTCGGGCTGCAAAAAA<br>CTGCATGGCGTGCAGGTACGCCTGCACGCGCTTCACTAACTTCATTGTTGAC<br>GACAGAGGACGCATACATCGGTGGAAGTCTCCTGTCGTCGTGGAAAAACTT<br>GGCAAGGCAGAGGTGGGAGGCAACTTGGTCACCATAAAGCACGTTATTCTT<br>GAAGGTGTGAAGGCTAAACCTTTGGCTAGAACTGCAGCTGAGCAGTAGGCT<br>AGCAGATCTTTTT
PRRSV-2 (01NP1)<br>Accession# AAP57172.1	TATCACATGCACCGGTCTCGGTAAATGTCTTACCGCTGGCTGCTGTTCTCGCC<br>TTCCGTTTCTCTGGTGCATCGTCCCCTTTTGTTTTGCTGCTCTCGCTAATGCTTC<br>TAACAGCTCTTCCCATCTCCAGCTGATATACAACCTTACCATTTGCGAACTTA<br>ACGGTACGGATTGGCTCAAACATAACTTTGACTGGGCGGTGGAGACCTTCGT<br>GATCTTCCCTGTGCTGACTCACATTGTGTCATACGGGGCACTCACTACTTCCC<br>ATTTCTTGGACGCAGTTGGTCTCATCACAGTCTCAACCGCAGGTTACTACCAT<br>GGACGGTATGTTCTCAGTTCTATCTACGCTGTTTGCGCACTTGCTGCGTTGAT<br>ATGCTTCATTATTAGGCTTACGAAGAATTGTATGAGCTGGCGGTACTCTTGCA<br>CCCGCTACACGAATTTTGTCTTGGACACAAAAGGAAAATTGTACCGCTGGA

	GGAGCCCAGTGATTATCGAGAAGGGCGGTAAAGTTGAGGTCGGTGGGCATC<br>TGATTGACCTTAAACGGGTTGTTCTCGACGGTAGTGCGGCCACCCCGGTCAC<br>TCGGGTTTCAGCAGAGCAGTGGGGTAGGCCATAGGCTAGCAGATCTTTTT
PRRSV-2 (NADC30)<br>Accession# AFP43978.1	TATCACATGCACCGGTCTCGGTAAATGTCTCACAGCCGGGTATTGTTCACAG<br>CTGCCGTTTCTCTGGTGTATCGTGCCGTTCTGCTTCGCAGCATTGGTGAATGC<br>TAATTCCAACTCTAGTAGCCATTTGCAACTTATTTACAACCTCACAATCTGTG<br>AATTGAATGGAACAGACTGGCTTAATGAAAGGTTCTATTGGGCCGTCGAGA<br>CATTTGTTATTTTCCCTGTCCTGACCCACATAGTTTCCTATGGTGCTCTTACGA<br>CATCACATTTCCTGGACACGGTTGGGCTGATTACGGTTTCCACGGCAGGCTA<br>TTACCACAGGCGCTACGTCCTCTCATCTATCTATGCCGTTTGTGCGCTGGCAG<br>CTTTGATTTGTTTTGCGATTCGCCTCGCAAAGAACTGCATGAGTTGGCGCTAC<br>TCTTGCACAAGGTACACTAACTTTTTGCTGGACACAAAAGGTAAATTGTACC<br>GCTGGCGCTCTCCTGTCATAATCGAAAAGGGAGGCAAGGTGGATGTCGGGG<br>GGCACCTCATAGACTTGAAGCGCGTGGTGCTGGACGGTTCCGCAGCGACTCC<br>AGTTACCAAAATCAGTGCTGAACAATGGGGGAGGCCATGAGCTAGCAGATC<br>TTTTT
PRRSV-2 (PRRSV0000008<br>973)<br>Accession# ACG54454.1	TATCACATGCACCGGTCTGGGAAAATGCCTGACAGCCGGCTGCTGCTCCAG<br>GCTTCTGTCACTTTGGTGCATTGTCCTTTTCTGTTTCGCAGTGCTGGTGAACGC<br>CAACAATAGTTCTTCTTCCCACTCCCAACTTATATACAACTTGACTATCTGCG<br>AGCTTAACGGAACAAAATGGTTGGGTAACAACTTTAACTGGGCGGTTGAGA<br>CCTTTGTTATCTTTCCTGTCCTCACTCATATAGTTAGTTATGGTGCTCTTACTA<br>CCAGCCACTTTCTTGACACGGTTGGCCTGGTGACGGTCAGTACCGCTGGATA<br>TTACCATGGTCGGTATGTCCTTAGCAGTGTTTACGCTGTCTGTGCCTTGGCGG<br>CGTTGATCTGTTTCATTATACGGTTCGTTAAAAACTGTATGTCTTGGAGATAT<br>TCATGTACGCGGCACACCAATTTTCTTTTGGATACGAAAGGACGCCTGTACA<br>GGTGGCGCTCACCTGTGATCGTCGAGAAGAACGGAAAAGTCGAGGTCGGAA<br>ACCATCTTATCGATCTGAAGAGAGTCGTGCTCGACGGCAGCGCGGCGACAC<br>CGCTTACGCGCGTCAGTGCAGAGCAATGGGGAAGACCCTGAGCTAGCAGAT<br>CTTTTT
PRRSV-2 (XW008)<br>Accession# AHC53194.1	TATCACATGCACCGGTTTGGGCAGGTGCCTGACCGCTGGGTATTGTTCAAGA<br>CTTCTGAGCCTCTGGTGCATCGTTCCTTTCTGGTTTGCCGTGCTCGTTAACGCC<br>AATAATACGAGCTCATCTCACTTCCAGTTGATCTATAACCTGACACTGTGTG<br>AGTTGAACGGCACCGAATGGCTTGGGAACAAATTCAACTGGGCAGTGGAAA<br>CCTTCGTGATATTCCCAGTGCTCACACATATAGTTTCCTATGGGGCGCTGACA<br>ACCAGTCACTTTTTGGACACAGTCGGCCTCGTGACCGTTTCTACAGCTGGCTT<br>CGTTCACGAACGCTACGTTCTTTCTTCAGTGTACGCGGTGTGCGCGCTCGCTG<br>CACTCATTTGTTTCACTATACGGTTGGCTAAGAATTGCATGAGTTGGCGGTAC<br>AGCTGCACCAGGTACACTAACTTCCTGCTCGACACCAAAGGGAAACTGTAT<br>AGATGGCGGTCACCTGTGATTATTGAGAAAGGTGGAAAAGTCGAGGTGGAG<br>GGACATCTCATCGACTTGAAGCGCGTCGTCCTGGACGGGTCAGCCGCTACTC

	CGCTGACGCGCGTTTCCGCAGAACAGTGGGGAAGGCTCTAGGCTAGCAGAT<br>CTTTTT
PRRSV-2 (GXLA12-2013)<br>Accession# AGL08390.1	TATCACATGCACCGGTCTTGGTAAATGTAGTATTGCAGGCTATTGCAGTCAG<br>TCTCTTTTTTTGTGGTGTATCGTTCCTTTCTGCTCAATAGCACTCGTTTCCGCG<br>AACGGAAACAGCTCATCTTATTCCCAGTTGATTTATAATTTGACCTTGTGTGA<br>GCTTAACGGCACCGACTGGCTCGCTGCTAAGTTTGATTGGGCAGTTGAATGC<br>TTCGTTATTTTTCCCGTGCTGACTCATATTGTCTCATATGGAGCCCTGACCAC<br>GAGCCATTTCCTGGATACGGTCAGCTTGGTCACAGTCAGTACCGCAGGGTTC<br>TATCACGGGAGGTACGTGCTCAGTAGCATATACGCAGTGTGTGCGTTCGCGG<br>CGTTTATCTGTTTTGTCATTCGCCTCGTCAAGAACTGTATGAGCTGGAGGTAC<br>TCATGTACCCGCTATACTAATTTTCTTCTTGACACAAAAGGGAAGATATACA<br>GGTGGCGCAGCCCAGTTATTATTGAGAAGGGAGGAAAGGTGGAGGTCGGGG<br>GTCACTTGATAGACCTCAAGCGCGTTGTCTTGGACGGTTCAGCGGCGACACC<br>TGTGACAAAGATTTCAGCAGAGCGGTGGGGTCACCCATAGGCTAGCAGATC<br>TTTTT
PRRSV-2 (MD001)<br>Accession# AKS03951.1	TATCACATGCACCGGTTTGGGTAACTGCCTGACAGTGGGATGCTGTTCAAGA<br>AGCCTGTTTTTGTGGTGTATAGTGCCTTTTTGCCTTGCAGCGTTGGTCTCCGCG<br>AATGGCAACAGTTCTTCTTATTCACAGTTGATCTATAACCTTACCTTGTGTGA<br>GTTGAATGGTACTGATTGGCTTGCCAAAAAGTTCGACTGGGCAGTGGAAACC<br>TTTGTGATTTTTCCCGTTATAACACACATCGTTTCCTACGGAGCCCTCACCAC<br>GAGTCACCTTCTGGATACTGTCGGATTGGTCACCGTTTCTACAGCGGGTTTCT<br>ACCACGGAAGGTATGTGCTCAGTAGTATCTATGCGGTCTGCGCGTTGGCAGC<br>ACTCATCTGTTTTGTCATCCGGCTCGCCAAGAATTGTATGTCTTGGCGCTATT<br>CCTGCACACGCTATACTAACTTTTTGCTTGATACGAAGGGCAGAATATATCG<br>GTGGAGATCTCCAGTTATTATCGAAAAGGGCGGCAAAGTGGAAGTCGAGGG<br>ACACCTCATTGACTTGAAGAGAGTGGTGCTCGACGGTTCCGCAGCAACTCCC<br>GTCACCAGGATACCGGCGGAAAGATGGGGGAGGCCATAAGCTAGCAGATC<br>TTTTT
PRRSV-2 (EDRD-1)<br>Accession# BAG49673.1	TATCACATGCACCGGTCTCGGAAAGTGTCTTACCGCCGGGTGCTGTTCACGC<br>TTGCCATTCCTCTGGTGTATTGTGCCGTTCTGCCTGGCGGCACTCGTGAACGC<br>CAGCGACAGCTCATCCTCCCACCTTCAACTCATTTACAACTTGACTCTGTGCG<br>AACTCAACGGTACGGATTGGCTCGCGGACAAGTTCGACTGGGCCGTGGAGA<br>GTTTTGTTATATTCCCCGTTCTTACGCACATAGTTAGCTATTGCGCATTGACG<br>ACTTCACACTTCCTCGATACAGTCGGCCTGGTTGCAGTTTCCACGGCCGGTTT<br>TTACCACGGGCGGTACGTGCTTTCTTCTATATATGCTGTTTGCGCACTTGCAG<br>CTCTGGTCTGCTTCGTCATAAGATTGACGAAGAATTGCATGAGCTGGCGGTA<br>CTCCTGCACCAGGTACACAAACTTTTTGTTGGATACAAAGGGTAGATTGTAT<br>AGATGGAGGAGCCCGGTCATTATTGAGAAGGGTGGTAAAGTCGAGGTGGAG<br>GGGCACCTCATTGATCTGAAGCGGGTTGTGCTGGACGGATCTGCAGCAACA<br>CCCATTACGAAGGTCAGCGCAGAGCAATGGGGCCATCCGTGAGCTAGCAGA<br>TCTTTTT

PRRSV-2 (Miyagi08-2)<br>Accession# BAJ14526.1	TATCACATGCACCGGTCTGGGCAAGTGTCTCACTGCCGGATGCTGTCTGAGA<br>CTCCCTTTTAGTTGGTGCATTATGCCCTTTTGTCTTGCTGTGTTGGTCAACGCG<br>AATGGCAACTCAAGTAGCCACCTTCAGCTTATTTACAATTTGACTCTTTGCGA<br>ACTTAACGGTACGGACTGGCTTGCTGGAAACTTCGACTGGGCAGTGGAGAG<br>TTTTGTTATTTTTCCTGTCCTCACCCATATAGTTTCATATGGGGCACTTACGAC<br>ATCCCATTTCCTTGACACGGTGGGACTTGTCACCGTTTCAACCACTGGTTTTT<br>ACCATGAAAGGTATGTCTTGAGTAGTATCTATGCAGTGTGTGCCCTTGCTGC<br>GCTCATTTGCTTCGTCATTAGATTGACGAAAAATTGTATGTCCTGGAGGTACT<br>CATGCACGAGGTACACAAACTTTCTTTTGGATACTAAAGGTAGATTGTACCG<br>CTGGAGGTCACCTGTGATTATAGAAAAAGGTGGTAAGGCTGAAGTCGAGGG<br>GCATCTCATCGACCTGAAAAGGGTTGTTCTTGATGGGAGCGCGGCTACTCCC<br>ATAACGAAAGTCAGTGCTGAACAGTGGGGGAGACCTTGAGCTAGCAGATCT<br>TTTT
PRRSV-2 (RespPRRS vac cine)<br>Accession# AAD27656.1	TATCACATGCACCGGTCTTGAGAAGTGTCTCACCGCAGGATGTTGTTCCCAA<br>CTTCTTAGTCTTTGGTGTATAGTTCCATTCTGCTTCGCTGTCCTTGCTAACGCT<br>TCAAATGACTCCTCCTCACACCTCCAGCTCATCTATAATCTCACACTCTGTGA<br>GCTCAACGGCACAGACTGGCTGGCGAATAAGTTTGATTGGGCCGTGGAGTCT<br>TTTGTCATATTTCCAGTCCTGACTCATATAGTCTCCTACGGTGCGCTGACTAC<br>CAGCCACTTTTTGGATACCGTCGCGCTCGTTACCGTTAGCACTGCTGGATTTG<br>TTCATGGAAGGTATGTGTTGTCCTCAATATACGCGGTGTGTGCGTTGGCCGCC<br>CTCACCTGCTTTGTTATCAGATTCGCTAAAAATTGTATGTCATGGCGCTACGC<br>CTGTACACGGTACACGAACTTTCTCCTGGACACCAAGGGGGGGCTTTACCGC<br>TGGCGGAGCCCCGTCATAATAGAAAAGAGGGGCAAAGTCGAAGTGGAGGG<br>ACATCTTATAGACCTGAAGAGAGTCGTTTTGGATGGCAGCGTCGCGACACCG<br>ATTACTCGCGTGTCCGCTGAACAGTGGGGAAGACCTTAAGCTAGCAGATCTT<br>TTT
PRRSV-2 (Sar01/2013)<br>Accession# AMW91022.1	TATCACATGCACCGGTTTGGGAGAATGTCTTACTGCAGGCTGTTGTTCTCAGC<br>TTCTGAGCCTTTGGTGTATCGTCCCATTTTGTTTCGCTGTTCTTGCTAATGCTTC<br>AAACGACTCATCATCTCATTTGCAGCTGATCTATAACCTGACGTTGTGTGAGT<br>TGAACGGTACTGACTGGCTTGCGAACAAGTTCGACTGGGCTGTCGAGAGTTT<br>CGTTATCTTCCCAGTTTTGACTCATATTGTTTCTTACGGGGCATTGACTACTTC<br>CCATTTCCTCGATACCGTTGCTTTGGTCACGGTGTCAACAGCAGGATTTGTTC<br>ATGGTCGCTATGTGCTGAGCAGCATCTACGCGGTTTGTGCTCTGGCAGCCCTT<br>ACTTGTTTTGTTATCCGCTTCGCTAAAAATTGTATGTCATGGAGGTATGCGTG<br>TACTAGATACACCAACTTTCTGTTGGATACGAAAGGGGGTCTCTACAGATGG<br>AGATCACCCGTCATCATTGAAAAGCGGGGAAAAGTCGAAGTGGAAGGACA<br>CCTTATCGATTTGAAACGCGTTGTTCTCGACGGGTCCGTTGCCACCCCCATAA<br>CTAGAGTCTCCGCTGAACAGTGGGGTCGGCCATGAGCTAGCAGATCTTTTT
PRRSV-2 (VR2332)<br>Accession# ABU87666.1	TATCACATGCACCGGTTTGGAAAAATGTTTGACGGCCGGATGTTATAGTCAA<br>CTCCTCAGCCTTTGGTGTATTGTTCCATTTTGTTTCGCTGTTCTTGTGAACGCTT<br>CTAATGATTCAAGTAGCCATTTGCAGCTCATATACAACTTGACCCTCTGCGA

	GCTTAATGGAACGGACTGGCTCGCCAACAAGTTCGATTGGGCGGTCGAGTCT<br>TTCGTTATTTTTCCCGTGCTCACTCACATAGTCTCTTATGGGGCCCTCACTACA<br>TCTCATTTTTTGGATACTGTCGCCCTCGTCACCGTCAGCACTGCAGGCTTCGT<br>CCACGGAAGATACGTGCTCTCAAGCATATATGCCGTTTGTGCACTGGCAGCC<br>TTGACTTGTTTCGTTATCAGATTTGCGAAGAATTGTATGTCATGGCGCTATGC<br>CTGCACTCGCTATACTAATTTTTTGCTGGATACAAAAGGGCGGCTCTACAGA<br>TGGCGGAGCCCCGTCATAATTGAGAAACGGGGCAAAGTTGAAGTTGAGGGG<br>CACCTGATCGACCTGAAGCGGGTGGTGCTGGATGGCAGCGTGGCTACTCCG<br>ATCACGAGGGTTTCTGCAGAACAGTGGGGCAGGCCATGAGCTAGCAGATCT<br>TTTT
PRRSV-2 (Ingelvac ATP)<br>Accession# ABJ51873.1	TATCACATGCACCGGTTTGGGCAGGTGTTTGACTGCTGGATGCTGCAGCCGC<br>CTTCTGTCCCTGTGGTGTATCGTCCCATTCTGCTTTGCTGCCTTGGTGAATGCT<br>AACTCTAACTCTTCTTCCCACTTGCAGCTCATTTACAATCTGACATTGTGTGA<br>GCTGAACGGCACGGATTGGTTGAAGGACAAATTCGATTGGGCTCTTGAGAC<br>CTTTGTGATCTTTCCCGTCCTCACACATATAGTGAGTTATTCCGCGCTCACAA<br>CGTCTCACTTCTTGGATACGGTGGGGTTGGTCACGGTGAGCACTGCAGGTTTT<br>TATCACGGACGCTATGTTTTGAGCTCTATTTATGCCGTTTGCGCCTTGGCAGC<br>TCTTACCTGCTTCGTCATCAGACTGGCGAAAAATTGTATGAGCTGGCGGTAC<br>TCATGTACTAGGTACACTAACTTCCTCCTTGACACGAAGGGAAGATTGTACA<br>GGTGGAGGTCCCCCGTGATTATAGAGAAGGGAGGAAAAGTCGAGGTGGAA<br>GGCCATCTTATAGACCTCAAAAGAGTTGTCTTGGACGGATCAGTTGCCACTC<br>CTCTTACGCGCGTTTCTGCTGAACAGTGGGGTAGGTTGTAAGCTAGCAGATC<br>TTTTT
PRRSV-2 (P129)<br>Accession# AAM18563.1	TATCACATGCACCGGTTTGGGCAAATGTCTTACAGCGGGGTGTTGTAGCCGC<br>TTGTTGAGCCTCTGGTGCATCGTGCCTTTCTGTTTTGCCGTCCTCGGCAGTGCG<br>AACTCTAGCTCAAGTTCTCACTTTCAACTCATTTATAACCTTACCCTGTGTGA<br>GTTGAATGGCACAGACTGGTTGGCCGAGAAATTTGACTGGGCAGTCGAGAC<br>ATTTGTGATTTTTCCAGTGCTCACACATATAGTCTCCTATGGTGCGTTGACCA<br>CTTCACATTTTCTGGACACCGTCGGCTTGGTGACAGTCAGTACAGCGGGATT<br>CTACCACGGAAGATATGTTCTGTCTTCCATCTATGCTGTCTGTGCGCTTGCCG<br>CACTCATTTGTTTCGTCATCAGGTTGGCCAAAAATTGCATGTCCTGGAGGTAT<br>TCTTGTACTCGCTACACTAACTTTCTGTTGGATACGAAAGGACGGTTGTATAG<br>GTGGAGAAGCCCAGTGATTATCGAAAAGGGTGGCAAAGTCGAGGTGGAAG<br>GGCACCTCATTGACCTTAAGCGCGTCGTCCTTGATGGATCCGTGGCTACTCC<br>GCTGACTAGGGTTTCCGCTGAACAGTGGGGAAGGCTGTGAGCTAGCAGATC<br>TTTTT
PRRSV-2 (Prime Pac)<br>Accession# ABG76930.1	TATCACATGCACCGGTCTCGGAAAATGTTTGACAGCCGGGTGCTGTAGTAGA<br>CTGCTGTCATTTTGGTGTATTGTCCCTTTTTGCTTCGCCGTCCTCGTGAATGCC<br>AGCTACAGCAGCTCCTCCCATCTTCAGCTCATATACAACTTGACACTGTGCG<br>AGCTCAATGGGACCGATTGGTTGGCAAACAAATTTGACTGGGCGGTCGAGA<br>GTTTCGTTATATTTCCAGTGCTGACTCACATTGTGTCCTATGGAGCCCTCACA

	ACTTCCCATTTTTTGGATACTGTCGGCCTTGTGACGGTCTCAACGGCCGGTTT<br>CTACCACGGCAGATACGTGTTGAGTAGTATCTACGCTGTGTGTGCGTTGGCT<br>GCCTTGATATGTTTTGTGATCAGGCTGGCGAAAAATTGTATGAGCTGGCGGT<br>ATTCCTGCACTAGATACACCAATTTTCTCCTGGATACGAAAGGCAGATTGTA<br>TAGGTGGAGGTCCCCAGTTATTATTGAGAAGGGTGGCAAAGTCGAGGTTGA<br>ATCTCACTTGATCGACCTTAAAAGAGTTGTGCTGGACGGGTCTGCGGCCACG<br>CCACTGACTAGGGTCTCCGCTGAGCAATGGGGTAGACCTTAGGCTAGCAGA<br>TCTTTTT
PRRSV-2 (Neb-1)<br>Accession# ACE87854.1	TATCACATGCACCGGTCTTGGGAAATGTCTTACTGCCGGATGCTGTAGTAGG<br>CTGCTCAGCTTGTGGTGTATCGTTCCTTTCTGCTTTGCCGTGCTCGTTAATGCG<br>AGCTATTCATCATCATCCCATCTTCAGCTCATTTACAACCTGACACTCTGTGA<br>ACTGAATGGTACGGATTGGCTGGCAAACAAGTTTGATTGGGCAGTCGAGAG<br>TTTCGTTATCTTCCCGGTTTTGACTCATATCGTGTCATACGGCGCACTCACTA<br>CTAGCCACTTCCTTGACACGGTGGGCTTGGTGACGGTTTCAACAGCGGGCTT<br>CTATCATGGCCGGTATGTGCTGAGTTCTATATATGCGGTGTGCGCCTTGGCGG<br>CTCTGATTTGCTTTGTGATCAGGCTCGCAAAAAATTGCATGTCATGGAGATA<br>CTCATGTACCCGGTACACCAACTTTCTGCTCGACACAAAGGGACGCCTTTAC<br>AGGTGGAGATCACCTGTGATAATTGAAAAGGGTGGCAAGGTTGAAGTGGAA<br>GGGCACCTTATCGACCTCAAGCGCGTGGTTCTCGACGGGTCCGCAGCTACGC<br>CCCTCACGCGCGTCTCTGCAGAACAGTGGGGGCGGCCCTAAGCTAGCAGAT<br>CTTTTT
PRRSV-2 (CH-1R)<br>Accession# ACF93752.1	TATCACATGCACCGGTCTCGGTAAATACTTGACTACCGGGTGCTGTAGTAGA<br>CTTTTGTCTCTCTGGTGTATAGTTCCGTTTTGCTTCGCTGTTCTGGTCAATGCC<br>AACTCCAACTCCTCAAGTCAGTTTCAACTTATCTACAACCTTACGCTGTGCG<br>AGCTGAATGGCACGGACTGGCTTGCAAATAAGTTCGATTGGGCAGTCGAGA<br>CATTCGTTATTTTTCCGGTGCTGACCCATATAGTCTCTTACGGAGCCTTGACT<br>ACCAGCCATTTCCTGGACACAGTGGGCCTGGTTACGGTCTCTACCGCCGGGT<br>TTTACCATGGACGGTATGTGTTGTCTAGCATTTACGCTGTTTGTGCCCTTGCTG<br>CTCTGATCTGCTTTGTGATTAGACTTGCTAAGAATTGCATGTCTTGGAGATAT<br>TCATGCACGCGCTATACTAATTTCCTTCAGGATACAAAAGGACGGCTGTATA<br>GATGGCGGTCACCAGTGATTGTTGAAAAGGGCGGAAAGGTTGAAGTTGAGG<br>GGCATCTCATCGACCTCAAACGGGTGGTCCTGGATGGGAGTGTGGCTACACC<br>TCTCACAAGAGTCTCAGCAGAGCAATGGGGAAGGCTTTAAGCTAGCAGATC<br>TTTTT
PRRSV-2 (HG.RV2)<br>Accession# AFM37611.1	TATCACATGCACCGGTCTTGGCAAATGCCTGACAGCATGTTGTTGTAGTAGA<br>TTGCTGTTCCTTTGGTGCATCGTCCCTTTTTATCTGGCAGTTCTTGCCAATGCG<br>AGCAATTCTAATTCATCACATATACAGCTTATTTACAATCTCACGCTCTGCGA<br>ACTTAATGGAACTGATTGGCTTGCTCAAAAATTTGATTGGGCTGTCGAAACT<br>TTCGTGATATTCCCCGTTCTGACGCACATCGTTAGTTACGGCGCCCTCACCAC<br>CAGCCACTTCCTGGATACCGTCGGGCTGGCCACTGTCAGCACGGCTGGATAT<br>TACCACGGCAGATATGTCTTGTCCTCTATATATGCTGTCTGCGCCCTCGCTGC

	ACTCATCTGTTTCGTTATAAGACTTGCGAAGAATTGCATGTCCTGGCGGTACAGCTGCACACGCTATACTAATTTCCTGCTGGACACTAAAGGACGCCTTTACAGATGGAGAAGTCCTGTCATCGTGGAAAAGGGGGGAAAGTGGAAGTGGAGGGTCACCTTATTGATCTCAAGAGAGTTGTTTTGGACGGCAGTGCGGCTACGCCTCTGACACGGGTTAGTGCTGAACAATGGGGAGACTCTAGGCTAGCAGATCTTTTT
PRRSV-2 (JXA1)<br>Accession# ABL60902.1	TATCACATGCACCGGTCTTGGGAAATGCCTCACTGCCTGTTGCTGCTCAAGGCTCCTGTTCCTCTGGTGTATCGTTCCGTTCTACCTGGCTGTCCTTGTTAATGCCTCTAATAACAATAGCTCCCATATTCAGTTGATATATAACCTTACTCTTTGCGAGTTGAATGGAACCGATTGGCTGGCACAGAAGTTCGATTGGGCCGTTGAAACTTTCGTGATATTCCCCGTTCTCACTCACATAGTGAGTTACGGAGCTCTGACGACATCACATTTCCTGGATACTGTGGGCCTGGCTACAGTTAGTACGGCTGGCTACTATCATGGTAGGTACGTGTTGAGCAGTATATATGCAGTTTGTGCACTGGCGGCGCTCATCTGCTTCGTGATAAGACTCGCCAAGAATTGCATGTCCTGGCGGTATTCTTGTACTAGGTATACGAATTTCTTGCTCGATACAAAAGGAAGACTTTATCGCTGGCGCTCTCCTGTGATTGTTGAGAAGGGGGGAAGGTCGAGGTCGAGGGCCACCTGATTGATCTCAAGCGCGTCGTCCTGGACGGGTCTGCGGCTACTCCTCTCACCCGCGTGTCAGCCGAATTGTGGGGAAGACTGTAGGCTAGCAGATCTTTTT
PRRSV-2 (2000-2454)<br>Accession# ACB41436.1	TATCACATGCACCGGTCTGGGGAGATGCCTTACGGCGGGCTGTTGTAGTCGGCTTTTGTCCCTTTGGTGCATTGTGCCGTTTTGGTTTGCCGTTCTGGTCGATGCAAATAGCAACTCCTCCAGTCACTTCCAGTTGATCTACAATTTGACATTGTGCGAACTTAACGGGACTGACTGGCTTGATAAAAAGTTCGACTGGGCCGTCGAGACCTTTGTGATTTTCCCAGTCCTCACGCACATCGTCAGCTACGGGGCTTTGACAACGTCTCACTTCCTTGACACTGTTGGCCTGGTTACCGTTTCAACTGCTGGATTTTACCACGGGCGGTATGTGCTTAGCAGTATCTATGCTGTTTGTGCTCTCGCTGCCCTGATCTGTTTTGTGATTCGGTTCGCTAAAAACTGCATGAGTTGGCGGTACTCCTGCACTAGATATACCAATTTTCTCCTCGATACAAAGGGTCGGTTGTACCGCTGGCGCAGTCCTGTCATAATCGAAAAGGGTGGAAAGGTTGAAGTCGAAGGCCACCTTATTGACCTTAAACGCGTTGTCTTGGACGGCAGTGTTGCAACTCCACTGACACGGGTTTCTGCAGAACAGTGGGGCCGCCCCTAAGCTAGCAGATCTTTTT
PRRSV-2 (MN30100)<br>Accession# ABU87642.1	TATCACATGCACCGGTTTGGGTAGATGTCTGACCGCGGGCTACTGCTCACGGCTTTTGAGTCTGTGGTGTATAGTCCCTTTTTGGTTCGCTGTCCTTGTGAATGCAAATTCTACCTCCAGCTCACATTTTCAGTTGATTTACAACCTCACTCTGTGCGAGCTCAATGGTACAGACTGGCTCGCCGGTAAATTCGATTGGGCAGTTGAAGCTTTCGTGATTTTTCCGGTCTTGACTCACATAGTTTCTTATGGAGCTCTCACTACGTCTCATTTTCTGGATACGGTTGGTCTGGTGACAGTCTCAACGGCTGGCTTCTGTCATGGAAGGTACGTGTTGAGCTCCGTCTATGCTGTTTGCGCCTTGGCGGCCCTGATTTGCTTTGTCATAAGATTCGCTAAGAATTGTATGTCCTGGCGGTATTCTTGCACCCGCTATACCAATTTCCTCCTCGATACAAAGGGCAGGCTGTATCGCT

	GGAGAAGCCCTGTTATTATCGAAAAGGGCGGGAAGGTGGAAGTCGAGGGA<br>CATTTGATCGACTTGAAGCGCGTGGTCCTCGACGGCAGCGTGGCGACTCCCC<br>TCACAAGGGTGTCCGCAGAACAATGGGGACGCCCCTGAGCTAGCAGATCTT<br>TTT
Lopma virus<br>Accession# QYL35076.1	TGACTACGCGACCGGTACCTGCTCTAAAAGCTGGGCACCCTGCAGTATGCG<br>GTTCGTGTCTTTGCTTTCCGTCTTGGTTTTTGCACCATTTACACAACGGAGGC<br>GTCTAATACTACGCTTGCAACCATTTACAACCTCACGTTGTGCCAATTTAATG<br>TGACGGATGTCTCTAACCATTTCGATTATGTCATCGAAGGTGCACTTATTTAC<br>CCTCTTGTGACCCATGCAATTAGCCATTACTTTCTTACCACAGCGTATTTCCTT<br>GATTTCGCGCCTTTGGCTGCTATAAGTATCGCTGCAATATACCAGAAGTTGT<br>ATGTCCTGGGCGCGATCCACGCCTTTATGGCGATTGTTGCTCTGATTTTGCTTT<br>GTCGCAGAGTGATACTTAATATCCTGGCACTCCGGTATGCTTGCACGCGCCA<br>TACCAATTTCATTTTGGACACCAAAGGATCTGTCCATCTGAACAAATCCCCT<br>GTGTTGATTTCAGACCAGTTTGGTGTTAGACTCAACAACGCACACATCCAGC<br>CAAAAATAGTCGTCTTTGACGGTATTAAGGCTCACCCTGTGAACACAAGCCA<br>GGCTGAAGAGTGGGCAGCTTAAGCTAGCAGATCTTTTT
RtMruf arterivirus<br>Accession# ALI16783.1	TGACTACGCGACCGGTAGACCGTCCTGTTTGTTTTGGTTGTCACTCCTGTTCA<br>TAGGATGGTCATGTCCCGTTTCAGTGGCGGCGAATAGTAATTCAAGTAGCAC<br>TCTCCAACTCATTTACAATATGACCCTGTGTGAACTCAATGGGACAGATTTC<br>CTTGCGAACAAGTTTGACTGGGCGGTTGAGAGTTTCGTGCTTTTCCCAGTGTT<br>TACACATATTGTCTCTAGAGGGTTTATGACAACGAGCCACCTCCTGGACACC<br>ATAGGACTCGCAACCGTTACTATCTCTGGGTACTGGCATCAGAGATATGTCC<br>TTAGTAGTATTTACGCTGTTTGTGCATTCGCCGCGTTTTGCTGTTTTCTTGTCA<br>GAGTTATTAAGAACTGTATGTCATGGCGGTACTCATGCACCAGGTTCACAAA<br>CTTTATTCTCGACACTAAAGGACACTTGTTCAGATGGAAGAGCCCCATACTG<br>GTTGAGAGGGCTGGTAAGGTCGAGGTCGATGGACACCTTATTGATGTTAAGC<br>ACGTGATAATTGAGGGGACTAAAGCCAAACCCGTGACCAGAGTTCCCGCCG<br>AACAGTGGGGTAGTGCATGAGCTAGCAGATCTTTTT
RtEi arterivirus<br>Accession# ATP66641.1	TGACTACGCGACCGGTAAATGTTATAGATCCTCAGCTGCGTCTGTTATGCGG<br>GCATTCTACTGGTGTCTGCCCTTCTTTCTCTACATAGGCTGGTGTTGCCCGTGC<br>GCTTCCGCTTCTAATGGCACAGGCTCCACCATGCAGCTTATATATAACATGA<br>CACTTTGCGAACTGAACGGAACGGATTGGTTGGTCGATAAATTCGACTGGGC<br>AGTGGAGAGCTTTGTCTTGTTTCCGGTGTTTACTCACATTGTGAGTAGAGGAT<br>TCATGACCACCTCACATTTGCTGGATTGGATTGCACTCACAACCATTGCTACT<br>GCCGGTTATTATCATTCCCGGTACGTGCTCAGCTCAATTTATGCGGTGTGTGC<br>TTTCGCTGCCTTTGTCTGTTTTCTTGTGCGCTTTATCAAAAACTGCATGTCTTG<br>GAGATATAGCTGTACGAGGTACACAAACTTTATCCTCGATACCAAGGGAAG<br>ACTGTTTAGGTGGAACAGTCCAATCATCATTGAGAAACAAGGTAAGGCTGA<br>TGTGGACGGGCAACTCGTCGACATCAAACACGTTGTTATTCAAGGAACGAA<br>GGCGAAACCGCTTACGCGCGTCGCAGCAGAGCAGTGGGGCTCAGGTTGAGC<br>TAGCAGATCTTTTT

LDV (Isolate p)<br>Accession# WKR37874.1	TGACTACGCGACCGGTAAGTGCCTCAAGAAGCTGGGGAGCGGTTGGATTCC<br>ATCTCGCCTTCTCCCCTTCTGTTTCATTCTCTATTTCTGAGTACTGAGAACGC<br>CTGCGCGGCTGGTAACTCATCTACTAAGAATCTGATATATAACTTGACCCTC<br>TGTGAACTTAATGTTACAGGATTTCAACAACATTTTGGATATGCCGTTGAAA<br>CCTTCGTGATTTTTCCCGCGTTGACGCATCTTATTTCTCTCAACTTTCTCACGA<br>CTGCTCACCTCTTGGATTTCCTTTCCCTTGGAATAGTGGCCGGTGGCGGCTAC<br>TGGCATAAGCAATATGTCATCTCCAGTATCTACGCCTCTTGTGCCCTTTTGGC<br>ATTCATATTTTTCTGCTGTAGGGCCGTTAGAAATTGTATGTCCTGGAGATACA<br>AGTGTACGCGGTTTACCAACTTCGTGTTGGACACAAAAGGAAAGGTCTACA<br>GAAATCGGTCTCCAGTGCTTGTTGAGCAGCACGGACGGGTTATGTTGCAAGG<br>TCACCCAATCGAGGTCAAGACCGTGGTGTTGGATGGGGTCAAAGCAGTCCG<br>GGCAAAAACTGTTCCAGCCGAGAAATGGAGGCTTAGGCTAGCAGATCTTT<br>TT
LDV (Neuro-virulent typ e C)<br>Accession# AAA74108.1	TGACTACGCGACCGGTGGGGATGGGTATAATTTGGGGTTCGGACGGTTCGTC<br>GGTATCATGACGTGCCTCAACAGGCTCGGGGGTTTTCTCATTCCTAGCTGGTT<br>GCTGCCATATTATTTTGTCCTTTACATTTTGTCCACAGAGAATGCCTGTGTGG<br>CAGGGGATTCCTCCACGAAGAACCTGATATATACTAGCACACTCTGTGAACT<br>GAATGTTACCGGATTCCAACAGCATTTCGGGTACGCCGTTGAAACATTTGTC<br>ATTTTCCCTGCGGTTACACATCTCATATCTTTGAAGTTCCTTACAACAGCACA<br>CCTGCTGGATTTCCTTTCATTGGGGGTCGTGGCGGGTGGAGGCTATTGGCATC<br>AGCAATACGTTGTGTCCAGCATTTACGCGTCCTGCGCATTGTTGGCTTTCATC<br>TTTTTTTGCTGTCGGGCTGTCAGAAATTGCATGAGTTGGAGATATAAGTGTAC<br>TCGGTTTACGAATTTTGTTCTCGACACCAAGGGCAGGGTGTTTCGGAATAGA<br>TCATCTGTTCTCGTTGAACAGCATGGAAAAGTTCTCCTCCAGGGACAACCCA<br>TAGAAGTGAAAACGGTCGTGCTCGACGGCGTTAAGGCCGTGCGGGCGAAAA<br>CGGTCCCCGCTGAAAAGTGGGAGGCCTGAGCTAGCAGATCTTTTT
RtClon arterivirus<br>Accession# APT40626.1	TGACTACGCGACCGGTCCAGCGTGTTGTAGCTATCTCCTTTTCTTCCTGTTCAT<br>TGGTTGGTCTTGTCCAGCAACCGTCGCAGGAAGTGGCAACAGTTCCTCTACC<br>TTGCAAAGCATATACAATCTCACCGTTTGCGAATTGAATGGAACGCAGTGGC<br>TTGCGACTCACTTCAGCTGGGCCGCGGAGACCTTCGTGCTTTACCCCGTCATT<br>ACTCATATAATTAGCCGCGGTTTTATGACAACGAGTCACCTTCTGGACGCGA<br>TTGGTCTTGTCGCAGTGGCGGCTAGTGGGTATCATCACGGACGGTATGTGCT<br>CAGCTCCGTCTATGCTGTCTGCGCATCTGCTGCTTTCGTGTGCTTTGTGGTTCG<br>CATGGTTAAGAATTGTATGTCCTGGCGCTATAGCTGTACACGCTATACAAAT<br>TATATTTTGGATACAAAGGGTAGAGTCCACAGGTGGCACAGCCCCGTTCTGG<br>TGGAGCGGCAAGGGAAGATTGATGTGAATGGGGATCTTATCGACCCGAAAC<br>ACGTCGTGATAGAGGGAGTGAAAGCTCAGCCAGTCGTTAGGGTTCCCGCCG<br>AACAATGGGGGCCTCGCTGAGCTAGCAGATCTTTTT
MgAV1<br>Accession# QHD57634.1	TGACTACGCGACCGGTGGCCACAGGCGCAACCTGTGTCACTGCCGCGCCTG<br>CAGTGTGGTTACAATGAAATGCTGGTCAAAGTCTGCGGAGCCTCTTACACGG<br>GCATGCTGCCTGTGGCTTTGCTCACTCCTTTTTATAGGCTGGTTCTGTCCAGGT

	TCAGTCGCGGCTGATGGGAACTCATCAAGTACGCTTCAACTGATTTATAATC<br>TTACCCTCTGTGAGTTGAACGGTACTGATTGGTTGGCAAACAAGTTCGATTG<br>GGCCGTCGAGTCTTTTGTTCTTTTTCCAGTGTTCACGCATATCGTCAGCCAGG<br>GATTCCTGACTACGTCCCATCTTCTTGACACCATTGGATTGGCTACAGTGGCA<br>ATCTCCGGGTACTGGCATTCCAGATACGTGCTTTCATCAATCTACGCGGTCTG<br>CTCCTTTGCCGCCTTTGTCTGTTTTTTGATTCGCATTGTTAAAAATTGTATGTC<br>ATGGCGGTACTCCTGCACCCGCTTCACCTCATACATACTGGATACAAAGGGT<br>CGGCTCTTTAGGTGGAAGTCACCCATATTGATAGAGCAGGCTGGGAAAGTC<br>GCGGTTGACGGCCACCAGATCGACGTCAAACACGTTATTATTGAGGGAACT<br>AAGGCCAAGCCTATCACGCGCGTGCCTGCTGAGCAGTGGGGACTTGCGTAA<br>GCTAGCAGATCTTTTT
SHFV (LVR 42-0/M6941)<br>Accession# AIL48195.1	TGACTACGCGACCGGTTATCTCTGTCTTGGAAGATCCGAAACGCCCCTTATA<br>GGTCTGTTCCGCACCAGTTCCACATCCATATCATGGTTTTATGTCCTGTTTTTT<br>GTTTCTATCACATTTAGTTCCACGGGTGCGAGCGAAAATAATACGGGTACGA<br>CATGGATTAGCATATCAAAGTTTCCATCTCTTCTGTTGTCTCAGATCATTTCC<br>CCATCATATATTGTCAATATCAGTGTCTGCGGAGCGTTCGACATACAGAACA<br>ACACCCACTGGTTTACGCCGTGCAACCTCAGTGTGCTCAATCACTCTGACTG<br>CCATACCTGTAAATCTGAACAGTCTAACCAAAGTCTCCTTTCCAATTGCAGT<br>ACGTGTTTCACGCACTTGTCTTCTTGTTTTTTGCATACATACACAGGACATCA<br>TATAAACAATACTAGGCTGCTGTTGGAAACATATCTCGCTGTCCCCCTGCTC<br>ACTCACCTCCTTTCTTACAAGTTTGCTACAACAGCGTCTTTCTTGGACTTTGC<br>GTTCTTCGCTGGGCTTTCAATTACTGCCTATCGCTACGTGAGTCCCGCGATAC<br>TCTTTTTTCTCCCGCTGGCACTGATATTTTCAGCAATATTTATTAAAAAACTG<br>GTTGTGAACTGCATGGCTCTTCGGTTTGCGTGGACAAGGCATACTAATTTCAT<br>TATTGATGACCGCGGACGCCTCTTCGTCAACCACGATGATGTGCTCATTTCC<br>GATCCTCAAGGCCTCCGGGTCGGGCCGCATAAGGTGCGGGCGGCCAAAGTT<br>ATCCTGGGTGGAAGGGAGGCCAACCTGTTGCGCCAAGCCCATGTTGAAGAG<br>TGGTCCTGGTAAGCTAGCAGATCTTTTT
SHFV (B11661)<br>Accession# AIY55127.1	TGACTACGCGACCGGTTACTTGTGCTTGGGTCGGTCTGAGACTTCCCTGATTG<br>GTCTTTTCCGCACCAGCTCCACTTCTATCTCATGGTTCTACGTGCTTTTCTTCG<br>TTTCTATCACATTCAGCTCTACAGGCGCATCAGAGAATAACACCGGCACCAC<br>GTGGATTTCTATTTCAAAATTCCCTTCACTGTTGTTGTCCCAAATCATCTCTCC<br>ATCCTATATAGTGAATATAAGCGTCTGTGGTGCTTTTGATATACAGAACAAC<br>ACTCATTGGTTTACACCCTGTAACTTGTCAGTCCTCAACCACTCCGATTGCCA<br>TACTTGCAAATCAGAACAGAGCAATCAGAGTCTGCTGAGTAACTGCTCTAC<br>ATGCTTCACGCATCTCTCAAGTTGCTTTTTGCACACTTATACCGGGCACCACA<br>TAAATAACACGCGCCTTTTGCTCGAAACCTACCTCGCCGTGCCTTTGCTTACG<br>CACTTGCTTAGCTACAAATTTGCGACTACGGCTAGTTTTTTGGATTTCGCTTTC<br>TTCGCAGGGCTCTCCATTACTGCGTATAGGTATGTCAGCCCGGCGATCCTGTT<br>TTTCTTGCCATTGGCCCTGATCTTTAGCGCCATTTTCATAAAAAAACTGGTTG<br>TGAATTGTATGGCTCTCCGGTTCGCCTGGACCAGACACACGAACTTTATAAT

	CGATGATCGGGGGCGCCTGTTTGTCAATCATGACGATGTTTTGATAAGCGAT<br>CCACAGGGACTTCGGGTGGGGCCGCACAAGGTTAGGGCTGCGAAAGTTATA<br>CTGGGAGGGAGAGAGGCAAATCTGTTGAGACAGGCTCACGTCGAGGAGTG<br>GAGTTGGTGAGCTAGCAGATCTTTTT
Free State vervet virus<br>Accession# YP_009249819<br>.1	TGACTACGCGACCGGTCTCAGCGCTTCTCTCTTGTTCTGTGTTCTTTTATGCT<br>CCAAGTGCGGTGCTCAACGTCAGCAGCCTCAAATGTTTCACTCTTTTCCAAC<br>CCGTCAGGCTTCATTCAGGCACTGCAAAAGACCCTGATCTCTGATTCCTACG<br>TGGTCAACATCAGTATCTGCGGTGCCCTGTCTATTCAGAACAATACACATTG<br>GTTCCAAGACCTCAGCACTTGTAACCATACGGGGGCCAACAACGAAACACG<br>CAACAAGACCGACAGTTTTCCCGTCACCTGCTTGATCCATAACTATACAGGG<br>GTTAACATCAATCACACACGCCTCGCTCTCGAGACCTATCTCGCGGTGCCTT<br>TGGCCACATATTTCCTGTCATTTTATGCAGCTACCACCGCAGCTTTTCTTGAC<br>TTCCTGTTCTGGCTTGGCCTTGGGTTGACGGCTGCTCATTATGCCTCCCCAGC<br>AGTTATTATTTACGCTCCCTTGGCGTTGATCTTCTTGGTGGTGTTTCTTAAAAG<br>AGTCATAATTAACTGCCTTGCTCTGAGGTATGCATGGACCAGACATACTAAT<br>TTCATAATAGATCAAAGCGGTCGGTTGTTCGTTAATCACGATGATTGTCTGAT<br>AGAACGGAATGGAAAAACCGTGCTCAACAACCAAGAGGTGAAAGTGGGAA<br>AGGTGATCCTCGGGGGACGGCTTGCTCATGGGATTAAAGCGACTCATGTTGA<br>GGAGTGGGGCTGGTGAGCTAGCAGATCTTTTT
Zambian malbrouck viru<br>s 1<br>Accession# ANB32510.1	TGACTACGCGACCGGTCTCCACGCCTCACTCCTCTCATGTGTTTTGCTCACGT<br>TGCTTATTCAATTCTCAAACGGAGCCAATTCCAATACATCCCTCTTCTCCTCT<br>CCGGCGGGGTTTATTTCAGCACTCCAGCAGACCTTGATCTCTGACTCCTATAT<br>CGTCAATATAAGCATCTGCGGTGCACTGGACATTCAGAACCAGACTCACTG<br>GTTCCAGGACTTCTCTCATTGCAAACCGTTGACAGCTACGAGTAACACAACC<br>ACCTCCTATCCAGTTTCCTGTGAAATACAGAATTACACCGGACAGTATATAA<br>ATCATACGAGACTTGCCCTTGAAACCTATCTCGCTGTCCCACTGGCGACGTA<br>CTACTTGAGCTTTTTCGCTGCTACTACTGCCGCTTTTCTTGACTTCCTTTTTTGG<br>CTCGGCCTCTCCCTTACCGCAGCACACTTTGCTTCACCAGCATTCATTGTTTA<br>CAGTCCTCTTGCAATTGTGTTCTTGGTCGTCTTTCTGAAACGGGCGATAACAA<br>ATTGTCTTGCGCTTAGATATGCCTGGACCAGGCACACTAACTTCATTATCGA<br>CCAGTCTGGTAGGCTGTTTGTGAATCACGATGACTGTCTGATAGAACGCAAT<br>GGAAAGACAGTTATTGGGAATCAAGAGCTTAAGGTCGGGAAAGTTATACTG<br>GGAGGGCGGCTGGCGCATGGAATCAAATCAACGCACGTGGAAGAATGGGG<br>ATGGTAAGCTAGCAGATCTTTTT
DeBrazza's monkey arter<br>ivirus<br>Accession# YP_009121781<br>.1	TGACTACGCGACCGGTAAATGCTATGTTCGGAGTGAAAGGAGTAGTACCGG<br>GACTCACATTACGAGCGCGCTTAGTACGTTTCTTTTGCTCTGTGCCTGCTGTG<br>TGAGCACTTTTAAAACTGCAAGTGCCACCGATGTTGGGGGGTTTAGTAGTAA<br>CAGCACATTGTGGAGCTCTTTCAAAAATCATATCATTAGTGATTCTTATGTCG<br>TCAACATTAGTATATGCGGCGCCCTTGATATATCTAATAACACGCATTGGCT<br>GACGCCTTGCAACTACTCTCAGTTTAAGCAAGATTGTCTCGATGGAAATGGG<br>ACATTTAAGAAGAACGAGAATAAGTGTAACCACTCCTCCTGCTTTCTTCAGC

	ACTACACCGGGCAGAACATTAACTACACTCGGGTGATTCTCGAAACGTATTT<br>GGCGACACCGTTGTTTACGCATCTCCTGTCCTACTATGCTGCGACTACGGCC<br>GCCGGGCTTGACTTTCTGTACTTCGCGGGCCTGGCTCTGACCGCTGTCTATTA<br>TCAAAGTCCAGCATTCTTGACGTTTAGCCCGCTCGCTCTCATTTTCCTGGTTGT<br>CTTTGTCAGACGGTTGGTCCTTAATTGTATGGCGCTGAGATACGCATGGACC<br>CGGCACACAAACTTCATCATAGATCAGAATGGAAGGCTCTTTGTTAACCACG<br>ATGACGTTCTCATAGCCGATAAAGATGGAGTGAAAGTTGGGAGTCAGAAAG<br>TGAAAGTCGCTAAGGTGATACTCGGGGGACGGGAAGCCTGCCTTCTTAGAC<br>AGGCACACGTCGAGGAGTGGACATGGTAGGCTAGCAGATCTTTTT
Pebjah virus<br>Accession# AKI29935.1	TGACTACGCGACCGGTTACATATCAGTCCTCTTGAGCTTGACCTTGCTTTTGT<br>CCTCTACCGTCACAAGATGCGTGTCTTCTGATGGCGATGTGAAGTCTTATGC<br>GCCCTCAAGTTGGATGAGCAAAGTTAAAGAGATGATTCTGGAACCAAGCTA<br>CGTTGTCAACATTTCCATTTGCGGAGCGTTTGATATTCAGAATAATACCCACT<br>GGTTCAGACCATGCGACAGTTTTAAACAAAATATCACAGGCAACTGTAATA<br>ACGAGACCGCTAGATGCTCCGCCTTCCTCAACAACCTTCCATGTATGCTGCA<br>CAATTACACTGGATCTCATATAAACCATACTCAGGTCCTTCTCGAAACCTAC<br>TTGGCGGTTCCGTTGTTGACTCATTTGCTCTCTTACTACGCGGCGACCACTGC<br>AAGTTTCCTCGATTTCACATTCTTTGGTATGCTTGCAGGTTCTGCATACTACTA<br>CTTCTCTCCCTCTTTTTTTTTTTCGCTCCCCTCGCACTCATCTTCTTGTGTATAT<br>TTCTGAGGCGGATCATAACTAACTGTATGGCTCTTCGGTATGCGTGGACGCG<br>CCATACCAATTTCATAATAGATCAGAAGGGCCGGTTGTTCGTCAATCACGAC<br>GATGTTCTTGTGAGCGATCAAGGCCAAGTGAAATTGGGAGATCATCGGGTTG<br>TTGTTGCCAAAGTCATTCTCGGTGGAAGGGAAGCCCAACTTCTCCGGGAAAG<br>TCATGTGGAAGAATGGGCATGGTGAGCTAGCAGATCTTTTT
Mikumi yellow baboon virus 1<br>Accession# YP_009067059.1	TGACTACGCGACCGGTAACAAGTGCTATAGAAATTGGGAGCCGTTCTCCATG<br>CCATCACCGTCCACACTCAGCCTTTGCACCTTTCTGCTGCTTTTGACATCTTCT<br>TTGTCAGACATGGCGGATAATGCCACTTCTCATGATCCAGTCACCGTCTGGT<br>CCGACATATTCAGCAACCTTATGAGCCCTTCCTATGTTGTGAATATATCTATT<br>TGCGGGGCAGTGTCCATACAGAATGATACACATTGGTACAAACCATGCACA<br>GCCGCAGAGATTGGTAACGCCACTAAGAACGATGCAAACTACACCAAGGTG<br>AGTAGCCTGCCCTGTATCCTGCACTCTTATACAGGAGTGCACGTGAACCATA<br>CAAAGGTGTTCTTGGAGACTTACCTGGCTGTCCCGCTGCTCACACACATGCT<br>GTCTTACTTCTGCGCTACGACGGCTGCTTTTCTTGATTTCACTTTCTTTGGAGG<br>CCTTGCGGGGGCGGCATATTATTATCATTCTCCTGCTCTTTTGCTGTACACAC<br>CCTTGGCCCTTATTTTCCTGGTTGTTTTTATCCGGCGCTTGACATTGAACATCC<br>TGGCGTTGAGATTCGCATGGACCCGGCATACCAATTTCATAATAGACCAGA<br>AGGGACGGTTGTTTGTGAACCATGACGACGTCTTGATAGAGGGACCAAACG<br>GTGTCAAACTGGCTGATAAGGAGGTTCGGGTCGCAACCGTCGTCCTGGGGG<br>GCCGCAAGGCACATCTTCTCCGCAGCGCCCATGCAGAAGAATGGTCCTGGT<br>AGGCTAGCAGATCTTTTT

Kafue kinda chacma baboon virus<br>Accession# YP_009222003.1	TGACTACGCGACCGGTCGGGTGTGCTACAAGAACTGGGCTGCTTCACTGATG<br>CCGAGTTTGATTACGACTAGTGTCCTGTCCTTTCTTCTCGTTTCTATTTGTGTT<br>AGTCCAGCGGCCTCAAGTGGGTCCGGTAATTTTGGACTGAGTCGGGACAATT<br>TTGGGAGCATATTCAAAAACTTGGTGACACCCAGTTATGTTGTGAACATTAG<br>CATTTGTGGTGCGCTTAGCATGCAAAATGCTACTCACTGGTTTATGCCTTGTG<br>ACGTTGCGAAACTGAGGGTCAATTGCACAAATTCCAGCGAATCCGAAAGTT<br>CAGATAAAGAGGGGTGCACAAGCGCGATAGACAAATTGAGTGCACACTGCT<br>CCATACATCACTACACCGGAATAAATATAAATCATACTAAACTCGCACTGG<br>AAACCTATGTCGCAGCACCATTGCTGACTCACATGCTTAGTTATTACTTTGGG<br>ACTACCGCTGCATTTCTGGACTTTCTCTTTTTTGGGGGCTTGTCCATCGCGGCT<br>TATGGTTATAGCTCACCGGCCTTCCTCCTTTATACCCCGTTGGCGGTGATATT<br>CATGGTGGTCTTCTGCAAAAAGATCATACTTAATTTCTTGGCATTGCGGTTCG<br>CGTGGACACGCCACACAAACTTTATTATCGACCAAAAAGGGAGACTTTTTGT<br>CAATCACGACGATGTGCTTGTTGAAGGGCCGAATGGCGTGAGGTTCGGCGA<br>CCAAGAGGTCCGGATAGCGACTGTTGTCCTGGGGGGCAGGAAAGCAAATCT<br>GCTTCGGACCGCTCATGCAGAGGAATGGAGTTGGTGAGCTAGCAGATCTTTT<br>T
Simian hemorrhagic encephalitis virus<br>Accession# AKC89298.1	TGACTACGCGACCGGTGGTTACCTGAGCAGCTTTTCTCTGCTGTCTTTCAGCC<br>AATTTCTGTGTTGCGTCTTCTCTTTTTGTTTCGAACTGCCAAACCCCGACATCC<br>ATGTTCAGATATATCTCAATTACACGACCTGCCACATGCAAGGCAGGATTCA<br>AGCAGGCCCCTCCGCCCTTGGGGCCTGCTCTACCTTCAAGCACGATCAGTTT<br>ACAGGACAATTTGAACCGCTCAATCTGAACTATAGCACAGCAGCTCCTGTTG<br>GAGCCCTTGTTTTGGGGCTCCAACTGTTCGGCCACAGGCACCAGTGTACCTTT<br>AACGGAAGTTATTACTGTTGCAAACAACCGTTTAATTCACCATCACCACTTC<br>AACACCATCTTCCCGTCCTTCCACATCTCATTTTGCTGGGCGCAGTGGCGGTG<br>ACGCATGGAATGCTGTCCGCATGTTTGGTGCAGGGACGGAGGTTTACAACTA<br>AGCTCGATTGAGCTAGCAGATCTTTTT
Praja virus<br>Accession# QYL35096.1	TGACTACGCGACCGGTATATGCTCCCTCTCATTGGGACCAAGGCCTATGTTT<br>GGGTCCTCATCATATCTCGGATTCCTGCTGCCAATACTTTTTTGTTCGGCAC<br>GTTTAGTGGCTCCCAGGCGAATGCAACGGTTGAAAAATTCCCATATGTGAAT<br>CTTACACTCTGTTCTTTCAATGAGAGTCATCATCAGTATTTCAAATACATGGT<br>CGAGGGATATGCCGTGGTTCCCTTGTTTACACACGTTATAGGTTTTGTGGCGA<br>TGACTACGGCTACTGCTATCGATACGCTCGCCTTTGGTTCTGCTGCTTACATA<br>ACATATGCACACGGGGAAAATCTGATTCTGTGTATCGTTTATGGACTGTGTG<br>GTCTTGCGGGATTCTTGTGCTTCTGTGTTAAAATCTTTCAAAATTTCAAGGCTT<br>TGCGCGTCGCCTGCACTCGGAGAACCAATTTTATTCTCTCCGATAAAGGACA<br>GGTCATCCCCTATAAGCATCCGTATCTGCTGGAAAAGGGCGGAAAAACCCT<br>GCTTCCTGATGGTACACTGGTCACTACTAAGGCGGTCGTCATTTCCGGCAGC<br>AAGGCGGTCGCAGGTTCTCCTATCACAGCCGAGTTGTGGCGGCCCTAAGCTA<br>GCAGATCTTTTT

African pouched rat arterivirus<br>Accession# AJG06163.1	TGACTACGCGACCGGTGCCGTCCCGTATTGCCATGCAGTTGGAGACCACAAT<br>CTTATGCGCGATAGGAATACACCTTGTTCAATGATTTGCTTGCGGGGGACGC<br>GGTACCTGAGTTCCATGCTCAGTAGTCTGCAATGGGTCGTTTTCTGCGTGATC<br>TGCGTTTGCTGTGCCAGCTCTGGGGTGGCAGCTTCTGGAAACCTCACGTCTGT<br>TACGCATACTTGGACTCATAATTTGACACTCTGTGCGTTGAATAACACAGAG<br>AATGCGACAAAACGCTTCCAACATGTTGTGGAGGCATTCGCGGTTGTCCCTC<br>TGGTGACACATGTGGTGTCATTGTTTTTCTTCACAACCGCGAGCTTCCTGGAT<br>GCCGCCGCCTTTGGTCTTGCTTCATGGTATACCTTCCAAGGCGATGCTATTGT<br>GCTTTGTGGGTTGTATGGGCTCTGCGGAGCAATAGCTCTGTTCATCGCCAGTT<br>GGCGGGCTGTCTTGAACTGCCTCGCCTTTAGGTATGCCTGTACGCGGCGCAC<br>AAACTTCCTCTTGACTGACAAAGGGGCGGTGTGCCCATTGCAAGAGCGGTA<br>CGTGGTGATGCAGGGAAGCCAGGCTGTTCTTCCTGGGGGTCAAAAAGTTACC<br>CCGAAAGCTGTTATTCTTGGAGGGCGGGAGGCCAAGTCACTTAACAGTATTA<br>CGGCTGAACACTGGAGCCCCTGAGCTAGCAGATCTTTTT
Hedgehog arterivirus<br>Accession# QRL06414.1	TGACTACGCGACCGGTTGTTTGAAATGTTTGCTGGGGTTGCCCCACACGTTG<br>ATGAGGGGATTCACCAGTAGCTTCGCAGTGTGGTGTTTTTCAATTCTTTTTAT<br>TGCGAGTGTCCCCGCCGGGGTCACCGCCTCAAACGCTACGAACTATAGGCTT<br>TTTACAAATCTCACCCTGTGCGCGTTGAATGGGAGCAGTAATGCATATTTTA<br>AGTACCTTTCTGAAGCTGTTTTCAGTGCCCCCATCCTCAGTCAAATCATCAAC<br>CAAAAGTTCAAAACTACAGCCTCTTTTATCGACACAGCCTGTTTTACCATAG<br>TTGCAGCTTTCCTGTTTGCCGACCATACCGCACGGCTTCTCGGCAGTATTTAT<br>GCATGTATTGCTTTGATGGCCTTGAGTAACTTCGCCTGGAGGTTCTTTAACAA<br>CTGTCGCACTTGGAGATACGCATGCACACGCTTTACGTCATTTATACTTACG<br>ACTAAGGGGGCCGTTATCCGGTACTCCTACCCGTATCTGCTGCACAAGCAAA<br>AACAGGTGCTCTTGCCTGATGGGACCATGGTTGAACCCAAGCACATTTTGTC<br>AGGGGGTCGGTTGGTTACATCCGATACGGGTATTGAGGCGGAATTGTGGGC<br>GTAAGCTAGCAGATCTTTTT
Olivier's shrew virus 1<br>Accession# ASA49508.1	TGACTACGCGACCGGTAAATGCCTGAATAATTCCGCTCGGCCGCTGACACTT<br>TGGTCTGCATTGCCATTCTTTTTCATATTCTTGTGGTGCGGTGGCGTGCAAGTG<br>GGGATCGTCGGGGCCAGCAACACAACCTCTCACAGTCTCAAGTATTACTTGA<br>TCCATAACATCACCCTCTGCGAATTGAATACAACCGCGCTGCCCGGGGGTTA<br>TTCCTTCACCTATGTCGAGGAGTATTGGGTGATCGCGCCATTCATTTGCTATA<br>TTATGGGCTTCACTGCAAAAACACTCAGCCTGTCCTGCGATCTCTTGGTGGTG<br>GGTCCATTGTTCGGGGTTGCCGCCCACATGAAAGCTTATTATTTGATGGTTAT<br>GCTTATTCCAACTTCTGCCATCCTTCTGGGAGCCTGGCTTTACCAGGTGATTA<br>TTACCTTCATGACATGGCGGCACGCCTGTACCCGCCACACTTCATTCGTCAG<br>GAGTAGTGACGGCCAACTGGGAAAAATTAACTCACATGTGCTGCTTGTGAA<br>GGGCGGTAAAGCTCTTACCAACAACGGCTGGGTCAAACCCGACCTTGTGGTT<br>TTGAAGGGGCGGAAAGCGGTTGAAACACACTCTGTGCCGTGTGACCACTAC<br>GCATAAGCTAGCAGATCTTTTT

EAV (ARVAC)<br>Accession# ACE82280.1	TGACTACGCGACCGGTTTGTCTATGATTGTGCTGCTCTTCCTTTTGTGGGGCG<br>CACCTTCACATGCCTATTTTTCATATTATACAGCTCAGAGATTCACTGACTTT<br>ACACTCTGCATGCTGACAGATAGGGGGGTTATAGCTAACTTGCTCAGATACG<br>ATGAGCACACCGCCCTCTACAACTGTTCCGCGTCCAAGACGTGTTGGTACTG<br>CACTTTTCCTGACGAAAAAATCATCACGTTCGGGACTGATTGCGACGATACT<br>TACGCGGTCCCCGTGGCTGAGGTCCTGGAGCAAGCCCATGGCCCCTACGGG<br>GTTCTGTTCGGAGATGTCCCGCCCTTCATATATTACGGAAGAGAATTTGGGA<br>TTGTCGTCCTGGATGTCTTTATGTTTTATCCGGTGCTGGTGCTCTTCTTCCTCA<br>GCGTCCTGCCTTACGCGACCTTGATTTTGGAGATGTGCGTCTCCATCCTTTTT<br>ATTATCTACGGAATCTATTCCGGTGCCTATCTCGCGATGGGAATTTTTTCAGC<br>TACACTTGCTATACACAGCATAGTGGTGCTGAGGCAGTTGTTGTGGCTTTGTC<br>TGGCTTGGCGGTACCGCTGCACTCTGCACGCATCTTTTATCTCTGCAGAGGGT<br>AAAGTGTATCCCGTTGATCCCGAGCTGCCTGTTGCTGCAGCAGGGAATCGCC<br>TCCTCGTGCCCGGTCGCCCCACCATAGACTATGCAGTGGCGTATGGATCAAA<br>GGTCAACCTGGTGAGACTCGGAGCGGCGGAAGTTTGGGAGCCCTAGGCTAG<br>CAGATCTTTTT
EAV (F20)<br>Accession# ABR92874.1	TGACTACGCGACCGGTCTCAGCATGATTGTCCTCCTCTTCTTGTTCTGGGGAG<br>TGCCCTCTCACGCCTATTTCTCATACTACACAGCCCAGCGGTTCACCGACTTC<br>ACCTTGTGTATGTTGACAGATCGCGGCGTGATTGCAAATCTGCTCAGATACG<br>ATGAGCATACCGCATTGTATAATTGCAGTGCCAGCAAGACCTGCTGGTATTG<br>CACATTCCTGGATGAGCAAATCATAACATTCGGGACTGGCTGTAACAACAC<br>ATATTCTGTGCCTGTTAGTACCGTCTTGGAACAAGCGCATGGCCCCTACAGC<br>ACGCTTTTTGACGACATGCCCCCATTCATTTACTATGGAAGGGAATTTGGTAT<br>TTTCGTCATGGATGTGTTCATGTTTTACCCCGTTCTCGTCCTCTTTTTCTTGTCC<br>GTGCTTCCGTACACCACTTTGATCCTTGAAATGTGTGTGAGCATATTGTTTGT<br>CGTCTACGGACTTTACTCTGGGGCGTATCTTGCCATGGGTATTTTTGCTACTA<br>CGTTGGTGGTCCATTCAATAGTGGTGTTGCGCCAACTTCTCTGGTTGTGCATG<br>GCCTGGCGGTATCGGTGTACCCTTCATGCCAGTTTCATTAGCGCGGAAGGCA<br>AAGTCTATCCAGTCGATCCAGGGCTTCCTATCGCGACACTCGGAAATCGCTT<br>GTTGGTGCCTGGGCGGCCAACGATCGATTATGCGGTGGCGTATGGGTCTAAA<br>GTTAACCTTGTCAGACTTGGCGCTGCTGAAGTGTGGGAACCCTGAGCTAGCA<br>GATCTTTTT
EAV (GB_Glos_2012)<br>Accession# BAQ56335.1	TGACTACGCGACCGGTCTCTTTATGATAGTGCTCCTGCTCTCATTCTGGGGAG<br>TTCCATCTCATGCCTACTTCTCATACTACACGGCACAGCGCTTTACAGATTTT<br>ACTCTCTGTATGTTGACGGATAGAGGTGTCATAGCCAACTTGCTCAGATATG<br>ACGAGCACACAGCGTTGTATAATTGCAGTGCGTCCGCCGATTGTTGGTATTG<br>CACGTTTCTCGACGAACAAATCATTACTTTCGGTACCGGGTGCAATGACACG<br>TACAGTGTCCCAGTCAGCGTTGTCCTCGAACAAGCGCACGGCCCATACAGC<br>GTCCTGTTTGACGATATGCCCCCTTTCATTTATTATGGGAGGGAATTTGGTAT<br>TTTTGTGATGGACGTTTTCATGTTCTACCCAGTTTTGGTGCTGTTTTTCCTTTCT<br>GTTTTGCCTTACGCTACATTGGTCCTGGAAATGTGCGTTTCAATCCTCTTTGTG

	ATTTACGGGATCTATTCTGGGGCTTATTTGGCTATGGGCGTGTTTGCTGCCAC<br>CCTGGTCGTTCACAGTGTGGTGGTTCTCAGACAATTGCTCTGGCTCTGTTTGG<br>CATGGCGGTACCGCTGCACCCTTCATGCGTCTTTTATATCCGCAGAAGGCCG<br>GGTCTACCCCGTCGACCCCGGTCTGCCTATTGCGACAGCAGGCAACAGGCTG<br>CTCGTTCCGGGCCGCCCGACTATAGACTATGCGGTCGCATACGGTAGCAAG<br>GTGAATTTGGTGAGACTCGGCGCGGCGGAGGTTTGGGAGCCTTAAGCTAGC<br>AGATCTTTTT
Wobbly possum disease virus<br>Accession# YP_009130637<br>.1	TGACTACGCGACCGGTAAGGCGCTGATTGACCTCACGTACGCCAACCTGAC<br>GATGGAACTGGGTCACCAGATCAACGTGAGAGAGGAGAATGGCACGTACG<br>CGCAGGCCGCGGCGCTGCTCTTTGCCAGCATCGCCTCAATGCCTCACTTCAC<br>GCTTGGTTCTCACCCGGTCAAGCGGGTCAGTGTCCTTTATCACCATAATAAG<br>ACCGACCACTGCTTCGGGGTCAGGCTTCACATCGCTACACACTTTTGTAACC<br>AGACGAAACTTGAGGTCAATTGCATAAATGACTACCTCCACACATGCCAAA<br>TCCCTCTTTGTGCGCATGGTAACCTTCTCTGCGTTCCCACCCATGTGAGGTGT<br>AATCCGTGGTTTGCTAGAACTTCCTTTTTCGATATGTACTTGCGCTCACTGGC<br>TCTTTCTGACAAGCAACGGTACCACAAGTATTTGGACTATCACGCGCACTTG<br>TCCTTTGCTGCCCCCATCACTTGTCTTATACTCACAACTTATGTGATCTTCACA<br>CTGATGGCTCGGGTGAGAATCTCTGGCTAAGCTAGCAGATCTTTTT
EAV (PLD76)<br>Accession# AAA92913.1	TATCACATGCACCGGTTTGTCTATGATTGTTCTGTTGTTCTTGCTGTGGGGAG<br>CTCCAAGCCACGCCTACTTCTCATATTATACGGCGCAGAGATTTACTGATTTT<br>ACTTTGTGCATGCTGACCGATCGGGGCGTTATCGCTAACCTCTTGCGGTATG<br>ACGAACATACGGCACTCTATAATTGTTCTGCGAGCAAGACATGCTGGTATTG<br>CACTTTTCCCGATGAGCAAATAATCACTTTTGGGACAGATTGCGATGACACT<br>TATGCGGTTCCCGTGGCTGAAGTGTTGGAACAGGCCCACGGCCCATACGGTG<br>TGTTGTTTGGAGATATGCCCCCTTTTATATACTATGGCAGGGAGTTTGGTATT<br>GTTGTTATGGACGTGTTCATGTTCTATCCAGTTCTGGTCCTTTTCTTTCTGAGC<br>GTCCTTCCGTATGCGACTCTCATTTTGGAAATGTGCGTTAGCATTTTGTTTATT<br>ATATATGGGATCTACTCCGGGGCTTACCTTGCTATGGGGATTTTTAGCGCTAC<br>CCTCGCGATACATAGCATAGTCGTTCTTAGGCAACTTTTGTGGCTTTGTCTTG<br>CTTGGCGGTATAGGTGCACTCTCCATGCCTCCTTCATATCTGCGGAAGGGAA<br>GGTTTACCCCGTCGACCCAGGGCTCCCAGTCGCAGCCGCTGGGAATAGGCTC<br>CTCGTTCCAGGTCGGCCTACCATTGATTACGCGGTGGCTTATGGTTCCAAAGT<br>GAACTTGGTTCGGTTGGGCGCTGCGGAGGTTTGGGAACCGTAAGCTAGCAG<br>ATCTTTTT

Supplementary Table S2. Synthesized DNA for generating plasmids encoding pig TRIF protein

pig TRIF<br>Accession#<br>NP_001302667.1	TTTTGGCAAAGAATTCGCCACCATGGCGAATACATCACCTTCTCTGAGCGGGGCATTT<br>AATATACTGTCCGCAGCCGGGCAAGGGAAGCTGTTGTATTTGAAGCACAAGCTCAAA<br>ACACTGAGGCTGGGATGTCAGGGGGCTGACTTGCTGCACGCAATGGTTCTCCTTAAAT<br>TGGGGCAAGAGACAGAGGCACGCATTTCCCTCGAAGCCCTTAAAGCCGACGCTGTTG<br>CTCAGCTCGTCGCGCGGCAATGGGCGGGCGTGGATAGTACTGAGACGCCTGAAGAGC<br>CCGCAGATGTTAGTTGGGCGGTCGCACGCGTCTACCACCTCTTGGTCGAGGAGAAACT<br>TTGTCCAGCCTCAATGCGGGAGGAAGCTTATGGCGCAGCACTCCGGGCCTTCAGGAG<br>TCGGGATGATCACCAGCTCGGAGAGTTGCAGGAGGAGGCAAGGGATCGGTGTGGAT<br>GGGATATCCTTCGGGACATGGAGGATGTTCAAGCGCTCCGCAGTGACCTGGGTTGTCC<br>TAGATTGTCCTCTGCGTTGCCCTCTCCTCCGCGCAGCCATCCACGGCCGATTGAAGATT<br>TGTCCGGCTGGTCACGCGGACATAGCTTGCGCTCCACGGGGAGCCCAGCTTCCTTGGC<br>CTCAAATCTCGAAATCAGTCAAAGCCCAACTATGGCGTTGCTGAGCCTGCATCACTCT<br>CCTCACGGTCCCTCAAAGTTGTGCGATGAACCGCGCGCTAGTCCAGTTCCGGAACCA<br>GCCCCTATGGGATGTCAGGAACCCGAAGAAATGAGCTGGCCGCCGTCTGTTGAGGGA<br>GCAAACTCTCCTGTGCAGAGTAATTCCCCCGTTCCTCGGGACCTGGAGGTGGCGGCAG<br>ATGCGAGCCCCGCGAGTCTCCCAGAAGCCCCAGAGGCGCCAGAGACAAGCACTCAT<br>TACCCCGTGGAATGTACGGAAGAGCCGGCCGCGCCGAAAAGTCTCCCTAGTCCTTCC<br>AGGAATGCGAGTCCGGATGTTACGGACCAAAAGCCACCGCTCCACTTGTCTGAAGAG<br>GATACCACGTATCCTACTGCGCAGCCACATCCTCCTACACCCTCAGTGCCCCAAACAT<br>CCCCGCCATTCCCGTCCCCGTCTACGCTCTCTAGCGCGCCACCTACGGTGAGTAACCC<br>TTCCCCGCCTGCCCCCGAACTCGAATTGAGCGAGCAGAAATTCTACAATTTTGTGGTG<br>CTGCATGCCGGCGCCGACGAACATATTGCCCTGAGGGTGAGGGAGAAGCTCGAATCT<br>CTTGGCGTTCGCGATGGTGCGACATTCTGTGAGGATTTCCAGGTCCCCGGTCGCGGTC<br>AGCTGAGGTGCCTTCAAGACGCTCTGGACCATAGCGCGTTTACCATCCTGCTGTTGAC<br>CCCCAACTTTGACTGTCGCCTGAGCCAACATCAAGCCAATCAATCCTTGATGAGCAGC<br>CTGACACGGCCAGGTTGGCAAGATTGTGTTATACCATTTCTCCCGTTGGAGTCCTCCCT<br>GGCACAACTCTCACCTGGTACGAGCAGCTTGTTGAGCTCACTGGTGTGGCTTGACGAA<br>CACAGCCAGATCTTCGCACGCAAAGTCGCCAATACCTTCAAACCGCAGAAACTTCGG<br>GCTAGAAAGGCAAAATGGCGCAAAGAACAAGACGCCAGAGCACTCCGCGAGCAGTC<br>CCAGCACCTCGAGGGCGAACGCCAACAGGCTGCGGCATGGGGTGCTGCCTGTTCAGC<br>TTATATACACAGCTACCTTTCTTACCAGACACAATTGGAAAAGCTGCAAATGGCCTTT<br>GCCAGTTACATGCCGTTCGGAGCCCAGCTCCCTAGTGCTCCCCCAGCTCCTTTCGGCG<br>AACAGGGACCGCTGGGCGCTCCACCGCCTTTTCCCACCTGGCCCGGTTTGCAACCCCC<br>TCCAGTTAGTCCCTGGTTGGCGGGAACACCTCCGCCGGCATTCCCCCAACCTCCGGCT<br>TTTCCTCAACCACCAGCGTTCCCTCAACCTCCTGCGTTTCCCCAACCACCCGCTGCAA<br>GCCAGTCCAGTCCTGTCAATCCACAGTCAAGTGGAGTCCAACCTTTGATAATCCACCA<br>CGCTCAGATGGTGCAGCTGGGACTGAATAATCATATGTGGAATCAAAGAGGGACTCA<br>GGCCCCGGAGGATAAGTCACCGGAAACGCAAGAGCAGAAGTTGATCTCCGAAGAAG<br>ATTTGTAAGCTAGCAGATCTTTTT

Supplementary Table S3. The oligonucleotides used to quantify ISGs mRNA levels

**Primer**	**Forward (5′ - 3′)**	**Reverse (5′ - 3′)**
Mx1	TACGACATCGAATACCAGATCAA	ATGGTCCTGTCTCCTTCGG
Isg15	GACTGCATGATGGCATCGGA	TGCACCATCAACAGGACCAT
β-actin	TGGATAAGCCTGCAGTCACAG	GCGTAGAGGTCCTTCCTGATGT
