# Supplementary material for: Conserved Antagonization of Type I Interferon Signaling by Arterivirus GP5 Proteins
Source: Viruses. 2024 Aug 1;16(8):1240. doi: 10.3390/v16081240 (PMC11358952; doi:10.3390/v16081240)
Supplement: Supplementary file 1 [file viruses-16-01240-s001.zip › 240727_Supplementary Figures Viruses_AS.pdf]

viruses

MDPI

A

**VR2332**	100%																					
**RespPRRS vaccine**	98.50%	100%																				
**P129**	90.50%	90.50%	100%																			
**01NP1**	85.92%	86.93%	88.44%	100%																		
**Prime Pac**	92.50%	92%	94.50%	89.44%	100%																	
**Ingelvac ATP**	90%	89.50%	94.50%	88.44%	92.50%	100%																
**JXA1**	87.50%	87%	91.50%	88.44%	91.50%	90%	100%															
**MN30100**	90.50%	90%	94%	85.92%	92.50%	92.50%	89%	100%														
**2000-5424**	90.50%	90%	95.50%	87.43%	93%	94.50%	90.50%	96%	100%													
**Neb-1**	93.50%	93%	95.50%	90.45%	99%	93.50%	92.50%	93.50%	94%	100%												
**CH-1R**	89.50%	89%	95.50%	86.43%	93%	93.50%	91.50%	92.50%	94%	94%	100%											
**PRRSV0000008973**	86%	85.50%	88.50%	88.44%	89.50%	86.50%	87%	88%	88.50%	90%	88%	100%										
**HG.RV2**	87.50%	88%	92.50%	89.44%	92%	89.50%	98.50%	89%	90%	93%	91%	87.50%	100%									
**NADC30**	84%	84%	87.50%	91.45%	87%	87.50%	86%	86%	87%	88%	86%	85.50%	85.50%	100%								
**GXLA12-2012**	82%	82%	85%	83.41%	84.50%	83.50%	83%	83.50%	84%	85.50%	83.50%	80.50%	82%	86%	100%							
**XW008**	87.50%	87.50%	91.50%	86.43%	90.50%	90.50%	88.50%	93.50%	92%	91.50%	90.50%	89%	88.50%	86.50%	82%	100%						
**MD001**	84.50%	84%	89.50%	85.42%	88.50%	88.50%	88%	87%	89.50%	89.50%	88.50%	83%	87%	85.50%	89.50%	85%	100%					
**Sar01/2013**	97.50%	99%	90.50%	86.93%	92%	90%	87%	90.50%	90.50%	93%	89%	85.50%	88%	84%	82%	88%	84.50%	100%				
**EDRD-1**	89%	88.50%	90%	89.44%	92.50%	90.50%	89%	88%	88.50%	93.50%	88%	84.50%	89.50%	87%	85.50%	85.50%	87.50%	88.50%	100%			
**GD**	88%	87.50%	92%	88.94%	92%	90.50%	99.50%	89.50%	91%	93%	92%	87.50%	99%	86.50%	83%	89%	88%	87.50%	89.50%	100%		
**Miyagi08-2**	87%	86.50%	89%	87.93%	91%	88%	88%	88%	88.50%	91.50%	88%	84.50%	87.50%	87%	84%	86%	87%	86.50%	92%	88.50%	100%	
**Zad-1**	54.50%	53.50%	54%	53.26%	53.50%	52.50%	54.50%	53.50%	53.50%	54.50%	54.50%	53.50%	55%	53%	51.50%	53%	53%	54%	53%	55%	52.50%	100%
	**VR2332**	**RespPRRS vaccine**	**P129**	**01NP1**	**Prime Pac**	**Ingelvac ATP**	**JXA1**	**MN30100**	**2000-5424**	**Neb-1**	**CH-1R**	**PRRSV0000008973**	**HG.RV2**	**NADC30**	**GXLA12-2012**	**XW008**	**MD001**	**Sar01/2013**	**EDRD-1**	**GD**	**Miyagi08-2**	**Zad-1**

B

**VR2332**	100%																					
**RespPRRS vaccine**	98.50%	100%																				
**P129**	91.50%	91.50%	100%																			
**01NP1**	89.94%	90.45%	91.95%	100%																		
**Prime Pac**	93%	92.50%	95%	93.46%	100%																	
**Ingelvac ATP**	92%	91.50%	96%	92.96%	94%	100%																
**JXA1**	90.50%	90%	92.50%	91.95%	94%	92.50%	100%															
**MN30100**	92%	91.50%	95.50%	90.45%	94%	93.50%	91.50%	100%														
**2000-5424**	92%	91.50%	96%	91.95%	94%	95%	92.50%	96.50%	100%													
**Neb-1**	94%	93.50%	96%	94.47%	99%	95%	95%	95%	95%	100%												
**CH-1R**	91%	90.50%	96%	90.45%	94%	95%	92.50%	94%	95%	95%	100%											
**PRRSV0000008973**	89.50%	89%	91%	91.45%	92.50%	90%	89%	91%	91.50%	93%	90%	100%										
**HG.RV2**	90.50%	91%	93.50%	92.96%	94.50%	92%	98.50%	92%	92%	95.50%	92%	89.50%	100%									
**NADC30**	89%	88.50%	92%	93.46%	92%	93.50%	90.50%	91.50%	92%	93%	91%	89.50%	90%	100%								
**GXLA12-2012**	85%	84.50%	88%	87.93%	87.50%	87.50%	87%	87.50%	87.50%	88.50%	87%	86%	86%	89.50%	100%							
**XW008**	90.50%	90%	94%	90.45%	93.50%	92.50%	92.50%	94.50%	93.50%	94.50%	93%	92%	93%	92%	86%	100%						
**MD001**	87%	86.50%	91.50%	90.45%	91%	91%	91%	89.50%	91.50%	92%	91%	87.50%	90%	90%	91.50%	88.50%	100%					
**Sar01/2013**	97.50%	99%	91.50%	90.45%	92.50%	91.50%	90%	91.50%	91.50%	93.50%	90.50%	89%	91%	88.50%	84.50%	90%	87%	100%				
**EDRD-1**	90%	89.50%	93%	94.97%	94.50%	93.50%	93%	91.50%	91.50%	95.50%	91%	89.50%	93.50%	93%	88%	90.50%	91.50%	89.50%	100%			
**GD**	91%	90.50%	93%	92.46%	94.50%	93%	99.50%	92%	93%	95.50%	93%	89.50%	99%	91%	87%	93%	91%	90.50%	93.50%	100%		
**Miyagi08-2**	87.50%	87%	90.50%	92.46%	92%	90.50%	91%	90%	90.50%	92.50%	90%	88.50%	90.50%	91%	86.50%	89.50%	90%	87%	93%	91.50%	100%	
**Zad-1**	66%	65%	66.50%	65.82%	65%	64.50%	66.50%	66.50%	66%	66%	66%	64.50%	67%	67%	64.50%	67%	65.50%	65.50%	65%	67%	64%	100%
	**VR2332**	**RespPRRS vaccine**	**P129**	**01NP1**	**Prime Pac**	**Ingelvac ATP**	**JXA1**	**MN30100**	**2000-5424**	**Neb-1**	**CH-1R**	**PRRSV0000008973**	**HG.RV2**	**NADC30**	**GXLA12-2012**	**XW008**	**MD001**	**Sar01/2013**	**EDRD-1**	**GD**	**Miyagi08-2**	**Zad-1**

C

**Lopma virus**	100%																								
**RtMruf arterivirus**	35.75%	100%																							
**RtEi arterivirus**	35.38%	70.98%	100%																						
**LDV (Isolate p)**	41.53%	49.22%	45.22%	100%																					
**LDV (Neuro-virulent type C)**	38.97%	50.77%	43.84%	83.91%	100%																				
**RtClon arterivirus**	36.78%	69.94%	62.17%	47.66%	48.70%	100%																			
**MgAV1**	35.38%	84.45%	67.48%	50.25%	45.32%	69.43%	100%																		
**SHFV (LVR 42-0/M6941)**	29.23%	25.38%	22.66%	24.62%	25.23%	23.31%	22.62%	100%																	
**SHFV (B11661)**	29.23%	25.38%	22.66%	24.62%	25.23%	23.31%	22.62%	99.64%	100%																
**FSVV**	33.33%	27.97%	29.06%	27.13%	26.63%	25.90%	24.43%	46.72%	46.72%	100%															
**Pebjah virus**	36.41%	28.49%	28.07%	32.66%	29.43%	28.49%	27.60%	54.43%	54.43%	50.81%	100%														
**MYBV**	35.89%	24.35%	29.06%	28.64%	27.57%	25.38%	22.17%	49.21%	49.21%	52.45%	54.03%	100%													
**KKCBV**	32.82%	25.38%	28.57%	30.15%	28.50%	25.38%	23.52%	48.50%	48.50%	51.63%	51.61%	60.93%	100%												
**SHEV**	11.53%	12.82%	11.53%	12.82%	10.89%	10.89%	12.17%	13.46%	13.46%	14.10%	15.38%	14.74%	16.66%	100%											
**Praja virus**	30.25%	31.08%	32.51%	33.66%	31.52%	31.60%	29.55%	22.16%	22.16%	23.64%	26.60%	26.10%	28.07%	12.82%	100%										
**APRA**	33.33%	30.05%	30.04%	30.65%	28.50%	30.56%	26.24%	27.07%	27.07%	22.70%	26.63%	25.76%	24.45%	12.82%	36.94%	100%									
**Hedgehog arterivirus**	28.71%	31.60%	31.52%	34.67%	30.24%	29.01%	30.73%	20.48%	20.48%	19.02%	23.90%	23.90%	19.51%	15.38%	34.97%	32.68%	100%								
**OSV1**	23.58%	25.38%	28.57%	28.14%	24.75%	24.87%	26.69%	17.96%	17.96%	23.30%	21.84%	20.38%	20.38%	11.53%	24.13%	21.35%	21.46%	100%							
**EAV (PLD76)**	18.46%	17.09%	16.74%	19.09%	18.69%	17.61%	14.47%	12.15%	12.15%	13.52%	14.91%	14.50%	13.72%	5.76%	14.77%	15.72%	15.12%	16.99%	100%						
**ZMV1**	30.25%	26.42%	25.61%	25.62%	26.16%	25.38%	22.62%	47.71%	47.71%	78.83%	48.54%	50.20%	48.54%	14.74%	22.16%	24.45%	16.58%	22.33%	12.03%	100%					
**DMA**	30.76%	25.90%	27.09%	26.13%	25.70%	25.38%	26.24%	52.45%	52.45%	51.63%	54.83%	50%	46.03%	15.38%	22.16%	24.45%	19.02%	21.35%	13.72%	51.86%	100%				
**EAV (ARVAC)**	18.46%	17.09%	16.74%	19.09%	18.69%	17.61%	14.47%	12.54%	12.54%	13.93%	15.32%	14.90%	14.11%	5.76%	14.77%	15.72%	15.12%	16.99%	98.43%	12.44%	14.11%	100%			
**EAV (F20)**	20%	17.61%	19.21%	20.10%	19.62%	19.17%	16.28%	12.54%	12.54%	14.34%	14.51%	14.90%	12.54%	6.41%	15.27%	14.41%	16.09%	16.01%	90.19%	13.69%	14.11%	88.62%	100%		
**EAV (GB_Glos _2012)**	19.48%	17.09%	19.21%	17.58%	18.22%	19.17%	16.74%	12.54%	12.54%	14.34%	13.70%	14.90%	13.72%	5.76%	15.27%	14.41%	15.60%	15.53%	89.80%	15.35%	14.90%	88.23%	92.94%	100%	
**WPDV**	8.98%	9.55%	8.98%	12.35%	11.79%	8.98%	10.67%	11.79%	11.79%	12.92%	10.11%	8.42%	13.48%	8.33%	8.98%	9.55%	9.55%	10.11%	8.98%	12.92%	9.55%	8.98%	9.55%	8.98%	100%
	**Lopma virus**	**RtMruf arterivirus**	**RtEi arterivirus**	**LDV (Isolate p)**	**LDV (Neuro-virulent type C)**	**RtClon arterivirus**	**MgAV1**	**SHFV (LVR 42-0/M6941)**	**SHFV (B11661)**	**FSVV**	**Pebjah virus**	**MYBV**	**KKCBV**	**SHEV**	**Praja virus**	**APRA**	**Hedgehog arterivirus**	**OSV1**	**EAV (PLD76)**	**ZMV1**	**DMA**	**EAV (ARVAC)**	**EAV (F20)**	**EAV (GB_Glos _2012)**	**WPDV**

D

**Lopma virus**	100%																								
**RtMruf arterivirus**	46.11%	100%																							
**RtEi arterivirus**	46.66%	78.75%	100%																						
**LDV (Isolate p)**	50.25%	61.13%	61.30%	100%																					
**LDV (Neuro-virulent type C)**	48.20%	64.76%	58.62%	87.93%	100%																				
**RtClon arterivirus**	47.15%	80.31%	71.50%	61.13%	62.69%	100%																			
**MgAV1**	47.17%	89.11%	75.36%	63.31%	58.41%	78.75%	100%																		
**SHFV (LVR 42-0/M6941)**	44.61%	42.48%	36.45%	39.19%	38.78%	39.37%	37.55%	100%																	
**SHFV (B11661)**	44.61%	42.48%	36.45%	39.19%	38.78%	39.37%	37.55%	99.64%	100%																
**FSVV**	45.64%	43%	43.34%	40.20%	38.78%	41.96%	40.27%	56.14%	56.14%	100%															
**Pebjah virus**	48.20%	42.48%	40.39%	44.22%	39.25%	40.93%	40.72%	64.91%	64.91%	59.42%	100%														
**MYBV**	48.71%	41.45%	39.90%	41.70%	39.71%	37.82%	37.10%	60.15%	60.15%	62.70%	61.69%	100%													
**KKCBV**	46.66%	41.45%	39.90%	44.72%	40.65%	37.82%	38.46%	60.82%	60.82%	61.47%	62.09%	71.48%	100%												
**SHEV**	20.51%	19.23%	17.94%	21.79%	19.87%	19.87%	20.51%	26.92%	26.92%	23.71%	24.35%	23.71%	25%	100%											
**Praja virus**	37.43%	45.59%	43.34%	43.71%	41.37%	44.04%	43.34%	33.49%	33.49%	36.45%	36.94%	35.46%	36.94%	21.15%	100%										
**APRA**	45.12%	40.93%	39.40%	41.70%	39.71%	42.48%	36.19%	41.48%	41.48%	36.68%	37.11%	34.49%	35.37%	23.07%	48.76%	100%									
**Hedgehog arterivirus**	36.92%	42.48%	44.33%	43.21%	40.48%	43%	41.46%	34.63%	34.63%	33.17%	35.12%	33.65%	32.19%	24.35%	42.36%	43.90%	100%								
**OSV1**	32.82%	36.78%	37.43%	40.70%	36.89%	38.86%	39.32%	31.06%	31.06%	33.98%	33%	31.06%	33.49%	16.66%	33.49%	33.49%	32.19%	100%							
**EAV (PLD76)**	28.20%	29.53%	28.07%	29.14%	28.97%	29.53%	28.95%	23.52%	23.52%	26.63%	26.20%	22.74%	23.13%	17.30%	22.66%	27.07%	23.90%	26.69%	100%						
**ZMV1**	42.56%	44.04%	40.88%	39.69%	40.18%	42.48%	38.91%	58.09%	58.09%	84.23%	60.99%	61.41%	60.99%	25.64%	35.46%	38.86%	32.19%	33.49%	25.31%	100%					
**DMA**	44.10%	41.45%	39.40%	39.69%	36.91%	38.34%	39.36%	60.37%	60.37%	59.42%	60.88%	57.42%	56.22%	26.28%	33.99%	34.93%	33.17%	32.52%	22.74%	60.99%	100%				
**EAV (ARVAC)**	28.71%	30.05%	28.57%	29.64%	29.43%	29.53%	29.41%	24.31%	24.31%	27.45%	27.01%	23.52%	23.92%	17.30%	23.15%	27.51%	23.90%	26.69%	98.43%	26.14%	23.52%	100%			
**EAV (F20)**	30.76%	32.12%	31.03%	32.16%	32.24%	32.64%	31.67%	24.31%	24.31%	27.04%	27.01%	23.52%	22.74%	18.58%	23.64%	27.07%	25.85%	26.69%	92.15%	25.72%	23.52%	90.58%	100%		
**EAV (GB_Glos_2012)**	30.25%	31.60%	30.04%	30.65%	30.84%	31.08%	31.22%	23.52%	23.52%	26.22%	25.80%	22.35%	21.96%	17.94%	23.15%	26.63%	25.85%	26.21%	92.54%	26.14%	23.52%	90.98%	95.29%	100%	
**WPDV**	18.53%	20.78%	19.10%	22.47%	23.03%	19.10%	22.47%	22.47%	22.47%	25.84%	21.34%	21.91%	24.71%	12.82%	17.97%	17.97%	15.73%	16.85%	16.29%	25.28%	21.34%	16.29%	17.41%	17.41%	100%
	**Lopma virus**	**RtMruf arterivirus**	**RtEi arterivirus**	**LDV (Isolate p)**	**LDV (Neuro-virulent type C)**	**RtClon arterivirus**	**MgAV1**	**SHFV (LVR 42-0/M6941)**	**SHFV (B11661)**	**FSVV**	**Pebjah virus**	**MYBV**	**KKCBV**	**SHEV**	**Praja virus**	**APRA**	**Hedgehog arterivirus**	**OSV1**	**EAV (PLD76)**	**ZMV1**	**DMA**	**EAV (ARVAC)**	**EAV (F20)**	**EAV (GB_Glos_2012)**	**WPDV**

Supplementary Figure S1

(A, C) Sequence identity of GP proteins. **(B, D)** Sequence similarity of GP5 proteins. Sequence identity and similarity were calculated by Sequence Identities and Similarities website (http://imed.med.ucm.es/cgi-bin/sias_new.cgi?jobid=1710329285, accessed on June 14, 2024).

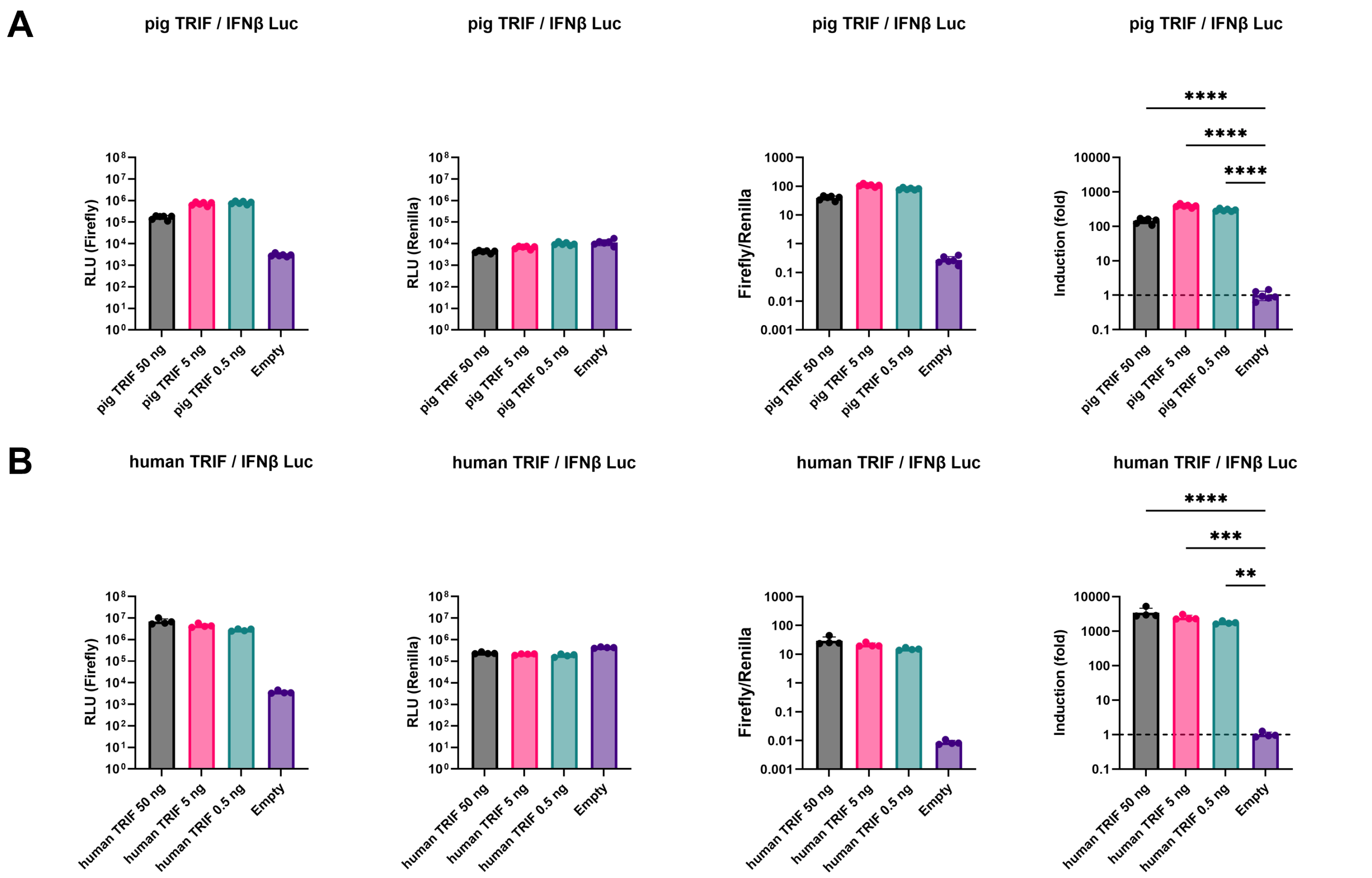

Supplementary Figure S2. Induction of Firefly luciferase.

Co-transfection of the following plasmids **(A)** IFN-β Luc and pig TRIF. **(B)** IFN-β Luc and human TRIF. Differences between IFN-stimulating plasmid and empty plasmid were examined by one-way ANOVA followed by Dunnett's multiple comparison test. ****$p < 0.0001$, ***$p < 0.001$, and **$p < 0.01$.

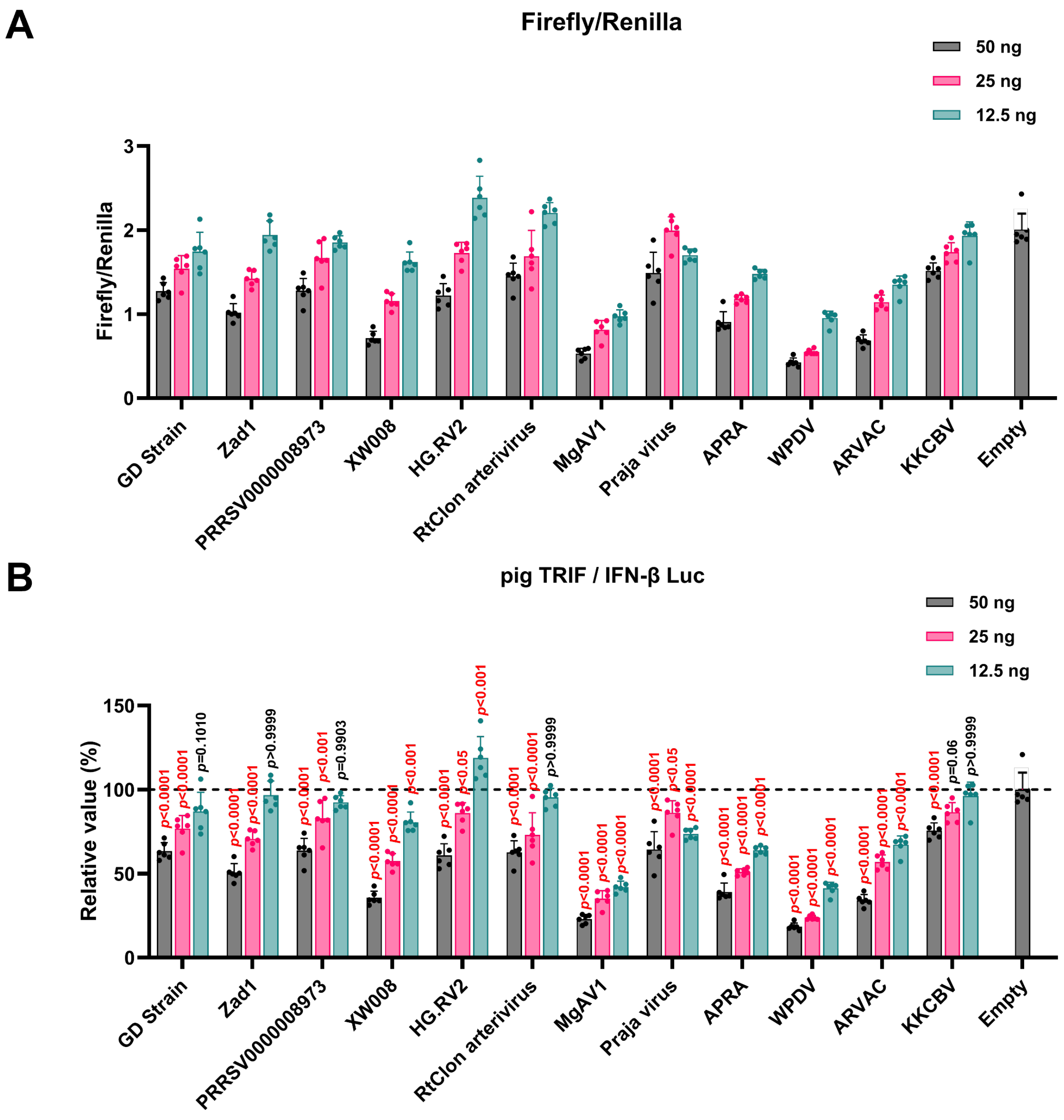

Supplementary Figure S3. Dose-dependent inhibition of IFN-β signaling by *Arterivirus* GP5 proteins. Lenti-X 293T cells were co-transfected with different amounts (50 ng, 25 ng, and 12.5 ng) of GP5 protein plasmids. (**A**) Raw data of the luciferase reporter assay. The RLU of Firefly luciferase was divided by the RLU of Renilla luciferase. (**B**) Relative value of IFN-β luciferase reporter assay. Differences between cells transfected with plasmids expressing *Arterivirus* GP5 proteins or an empty plasmid were examined by two-way ANOVA followed by Šídák's multiple comparison test.

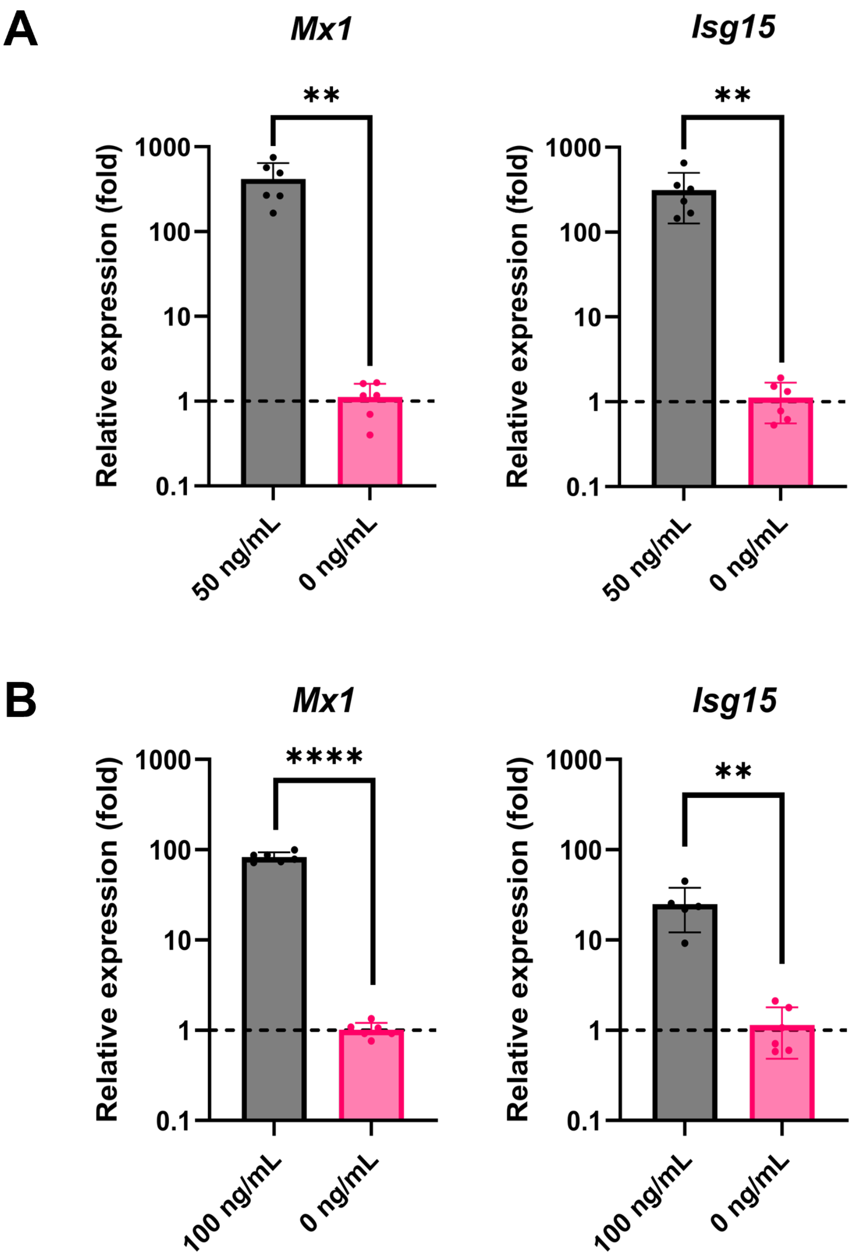

Supplementary Figure S4. IFN-stimulated genes (ISGs) induction triggered by Poly (I:C) or IFN-β.

Induction of ISGs mRNA in SK-6 cells triggered by poly (I:C) or IFN-β treatment. The results are presented as the mean and standard deviation of sextuplicate measurements from one assay. **(A)** Induction of ISGs mRNA triggered by 50 ng/mL poly (I:C) treatment in SK-6 cells. **(B)** Induction of ISGs mRNA triggered by 100 ng/mL IFN-β treatment in SK-6 cells. Differences between untreated cells and 50 ng/mL poly (I:C) or 100 ng/mL IFN-β treated cells were examined by two tailed, unpaired Student's t-test. ****$p < 0.0001$ and **$p < 0.01$.
